# Investigator choice of standard therapy versus sequential novel therapy arms in the treatment of relapsed follicular lymphoma (REFRACT): study protocol for a multi-centre, open-label, randomised, phase II platform trial

**DOI:** 10.1186/s12885-024-12112-0

**Published:** 2024-03-25

**Authors:** Graham McIlroy, Siân Lax, Charlotte Gaskell, Aimee Jackson, Malcolm Rhodes, Tania Seale, Sonia Fox, Lousie Hopkins, Jessica Okosun, Sally F. Barrington, Ingo Ringshausen, Alan G. Ramsay, Maria Calaminici, Kim Linton, Mark Bishton

**Affiliations:** 1 Cancer Research UK Clinical Trials Unit (CRCTU), University of Birmingham, Birmingham, UK; 2 National Cancer Research Institute, London, UK; 3 Division of Cancer Sciences, University of Manchester, Manchester, UK; 4 Barts Cancer Institute, Queen Mary University of London, London, UK; 5 King’s College London and Guy’s and St Thomas’ PET Centre, School of Biomedical Engineering and Imaging Sciences, King’s College London, King’s Health Partners, London, UK; 6 UCL Cancer Institute, University College London, London, UK; 7 School of Cancer and Pharmaceutical Sciences, Faculty of Life Sciences & Medicine, King’s College London, London, UK; 8 Department of Cellular Pathology Barts Health and Centre for Haemato-Oncology, Barts Cancer Institute, Queen Mary University of London, London, UK; 9 Department of Medical Oncology, The Christie NHS Foundation Trust, Manchester, UK; 10 Translational Medical Sciences, University of Nottingham, Nottingham, UK; 11 Department of Haematology, Nottingham University Hospitals NHS Trust, Nottingham, UK

**Keywords:** Clinical trial, Relapsed follicular lymphoma, Bayesian power prior methodology, Adaptive design, Epcoritamab, Lenalidomide

## Abstract

**Background:**

Relapsed or refractory follicular lymphoma (rrFL) is an incurable disease associated with shorter remissions and survival after each line of standard therapy. Many promising novel, chemotherapy-free therapies are in development, but few are licensed as their role in current treatment pathways is poorly defined.

**Methods:**

The REFRACT trial is an investigator-initiated, UK National Cancer Research Institute, open-label, multi-centre, randomised phase II platform trial aimed at accelerating clinical development of novel therapies by addressing evidence gaps. The first of the three sequential novel therapy arms is epcoritamab plus lenalidomide, to be compared with investigator choice standard therapy (ICT). Patients aged 18 years or older with biopsy proven relapsed or refractory CD20 positive, grade 1-3a follicular lymphoma and assessable disease by PET-CT are eligible. The primary outcome is complete metabolic response by PET-CT at 24 weeks using the Deauville 5-point scale and Lugano 2014 criteria. Secondary outcomes include overall metabolic response, progression-free survival, overall survival, duration of response, and quality of life assessed by EQ-5D-5 L and FACT-Lym. The trial employs an innovative Bayesian design with a target sample size of 284 patients: 95 in the ICT arm and 189 in the novel therapy arms.

**Discussion:**

Whilst there are many promising novel drugs in early clinical development for rrFL, understanding the relative efficacy and safety of these agents, and their place in modern treatment pathways, is limited by a lack of randomised trials and dearth of published outcomes for standard regimens to act as historic controls. Therefore, the aim of REFRACT is to provide an efficient platform to evaluate novel agents against standard therapies for rrFL. The adaptive Bayesian power prior methodology design will minimise patient numbers and accelerate trial delivery.

**Trial registration:**

ClinicalTrials.gov: NCT05848765; 08-May-2023.

**EudraCT:**

2022-000677-75; 10-Feb-2022.

**Supplementary Information:**

The online version contains supplementary material available at 10.1186/s12885-024-12112-0.

## Background

Follicular lymphoma (FL) is a common and incurable non-Hodgkin lymphoma [1]. Despite significant developments in first-line treatment [2], most patients still experience multiple relapses and increasingly shorter remissions over a long disease course and ∼ 20% have primary refractory disease characterised by early disease progression and death [3].

Outside trials, treatment of relapsed and refractory (rr)FL is limited to a handful of non-cross-resistant regimens. The most common treatment is rituximab in combination with a chemotherapy backbone of CVP (cyclophosphamide, vincristine and prednisolone), CHOP (cyclophosphamide, doxorubicin, vincristine and prednisolone) or bendamustine [4]. Bendamustine combined with obinutuzumab, a more potent anti-CD20 monoclonal antibody, is an option for patients progressing within six months of rituximab-based treatment [5]. A two-year maintenance antibody phase may prolong remission in responding patients but has not been shown to improve overall survival (OS) even in studies with follow up exceeding 10 years [6, 7]. Rituximab with lenalidomide is an effective alternative to chemotherapy [8] and currently the only novel, non-chemotherapy National Institute for Health and Care Excellence (NICE)-approved option for rrFL patients in England.

There are no available data to guide treatment choice and sequencing of treatment for rrFL. Pragmatic therapy decisions are based on patient age and fitness, prior therapy and length of remission, previous treatment tolerance, physician preference, and availability of trials. Patients experiencing early treatment failure, cumulative toxicity or progressive treatment resistance after multiple treatment lines soon exhaust the small supply of standard therapy options, thus creating a need for novel, safe and effective therapies to overcome treatment resistance, improve treatment tolerance and extend the therapeutic armamentarium.

There are many promising drugs in early clinical development for rrFL. To date few have been approved due to limited knowledge of their efficacy compared with standard options and place in treatment pathways, driven mainly by a lack of randomised trials.

The REFRACT trial is a prospective, investigator-initiated UK National Cancer Research Institute (NCRI) randomised phase II platform trial designed to accelerate approval of novel therapies through evaluation against standard investigator choice standard therapy (ICT). The first of three sequential novel arms is evaluating epcoritamab plus lenalidomide.

Epcoritamab (DuoBody-CD3xCD20, GEN3013) is a bispecific antibody designed to engage CD3 on T-cells and CD20 on B-cells to form a cytotoxic synapse for efficient killing of CD20-positive lymphoma cells. This engineered antibody does not require T-cell receptor specificity or act through antibody-dependent cell-mediated or complement-mediated cytotoxicity [9]. Pre-clinical studies demonstrated potent activity against xenograft and primary follicular lymphoma cells [9, 10], with early results of a first-in-human phase I/IIa trial (NCT03625037) demonstrating that subcutaneously administered epcoritamab is both convenient and safe, with predictable and mostly low grade, manageable toxicities including pyrexia, cytokine release syndrome (nearly all grade 1–2), and injection site reactions [11]. Among 128 patients with FL treated with epcoritamab at the 48 mg recommended phase 2 dose, the overall response rate (ORR) was 82% with a complete response (CR) rate of 63% [12]. In addition, interim results of an ongoing phase II trial of epcoritamab in combination with rituximab and lenalidomide in rrFL show that epcoritamab can be safely combined with rituximab and lenalidomide with a manageable toxicity profile and no new safety signals. Among 101 efficacy-evaluable patients, ORR was 97% and CR 86% [13].

### Design

#### Study design

**Fig. 1:**
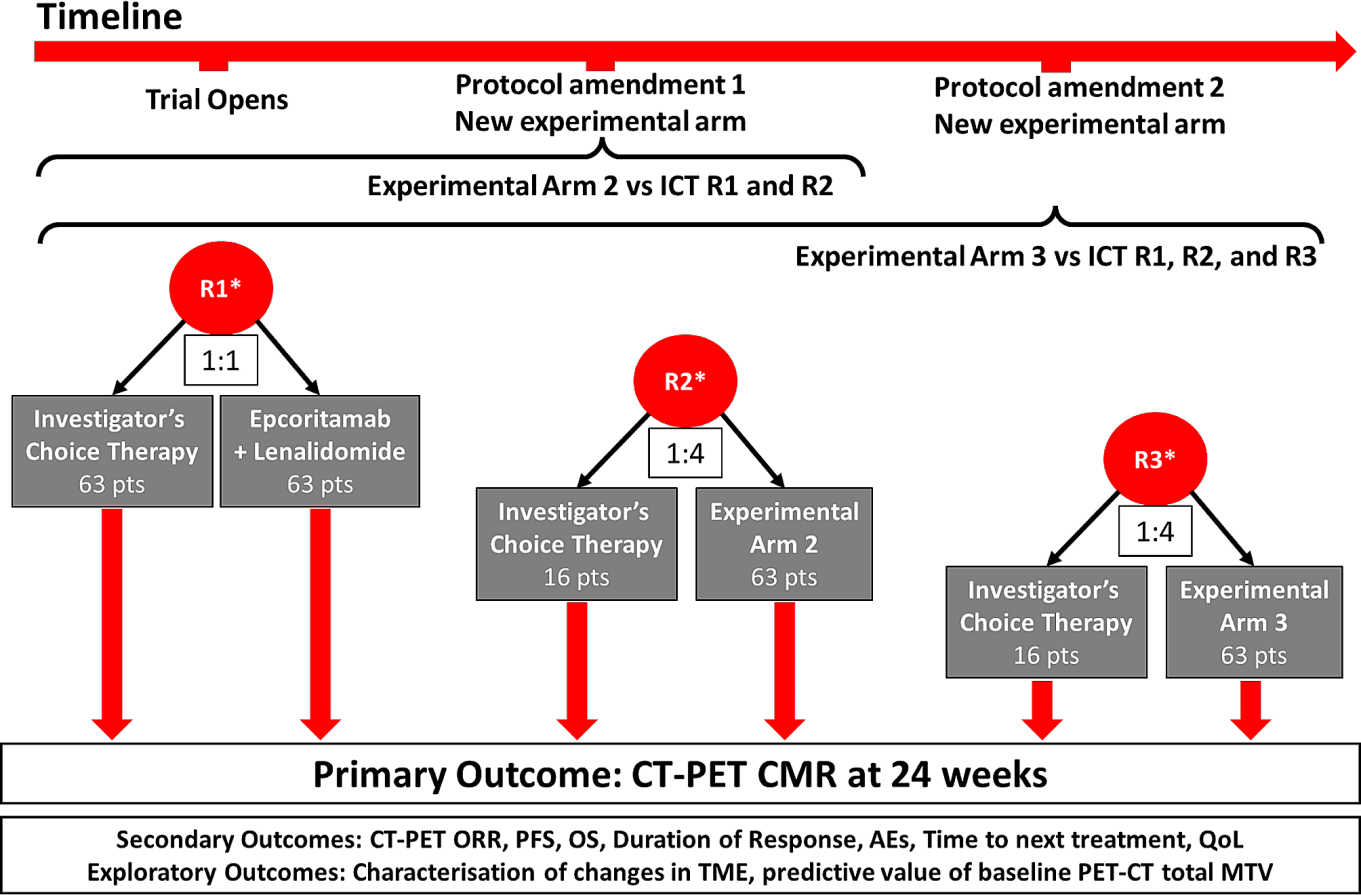
REFRACT trial schema. REFRACT trial schema showing the sequential three rounds of novel therapies (R1–3) which will be randomised and compared with investigator choice of standard therapy (ICT) in a ratio of 1:1 in R1 and 1:4 in favour of the novel therapy in R2 and R3. Patients receiving novel therapies in R2 and R3 will be compared with those patients who receive ICT in R1 and 2, or R1, R2, and R3, respectively. * Stratified by planned treatment regimen, consolidation intention, risk score, and number of lines of prior therapy. AEs, adverse events; CMR, complete metabolic response; ICT, Investigator choice of standard therapy; MTV, metabolic tumour volume; pts, patients; ORR, overall response rate; OS, overall survival; QoL, quality of life; R, round; TME, tumour microenvironment

REFRACT is a randomised, phase II platform trial for sequential evaluation of novel treatments versus ICT for rrFL. There are three treatment rounds; each has an ICT control arm and a novel treatment arm. In Round 1 (R1), 126 patients will be centrally, electronically randomised using a 1:1 allocation ratio to receive either ICT or epcoritamab + lenalidomide (novel treatment). In each of Rounds 2 (R2) and 3 (R3) (novel treatments yet to be determined), 79 patients will be randomised using a 1:4 allocation ratio in favour of the novel treatment (Fig. 1). A Bayesian data sharing technique will be used to provide adequate evidence on the control arm. Novel agents in R2 and R3 will be selected based on clinical drug development programs.

Patients will be stratified at randomisation by planned ICT regimen, consolidation intention, disease risk, and lines of prior therapy. No formal interim analyses are planned.

The trial will be delivered at up to 30 UK NHS sites, a list of which can be requested from the REFRACT Trial Office (REFRACT@trials.bham.ac.uk). The Standard Protocol Items: Recommendations for Intervention Trials checklist is provided as Supplementary Appendix 1 [14]. The World Health Organization Trial Registration Data Set is provided in Supplementary Appendix 2.

### Patient and public involvement

REFRACT was developed in collaboration with the EMERGE patient and public involvement (PPI) group and an NCRI low grade lymphoma patient representative (co-author MR). The EMERGE PPI group included eight FL patients at different points in their disease course, with a variety of treatment experiences and a mix of clinical trial experience. The EMERGE group is fully-funded, recruited from across the UK in collaboration with the Lymphoma Action charity, and has co-designed terms of reference. The PPI group and patient representative reviewed the trial proposal and advised on trial design, plans for sample collection and quality of life (QoL) outcomes. MR helped develop participant-facing documents and a trial summary for patients. As a member of the trial management group (TMG), he will assess study conduct, trial amendments, and support dissemination of the study results through existing advocacy activities and social media channels. An additional member of the EMERGE group has been recruited to the independent trial steering committee (TSC).

### Trial eligibility criteria

Eligible criteria are listed in Table 1.

**Table 1:** Key patient eligibility criteria for the REFRACT trial

**Inclusion Criteria**
Biopsy proven relapsed or refractory CD20 positive, grade 1-3a follicular lymphoma (biopsy within 6 months of trial entry)
Aged 18 years or over
Relapsed or refractory disease that in the opinion of the treating physician requires systemic therapy
Patient suitable for standard available therapy at the investigator’s discretion
Prior therapy with at least one line of immunochemotherapy. Previous radiotherapy at any time is permitted and will not count as a line of therapy. Previous rituximab monotherapy is also permitted as long as patients have at any time also received at least one line of immunochemotherapy
Assessable fluorodeoxyglucose (FDG)-avid disease by PET-CT [15]
ECOG performance status of 0, 1 or 2
Adequate organ function defined as; ANC ≥ 1.0 × 10^9^/L (growth factor use is permitted) Platelet count ≥ 75 × 10^9^/L, or ≥ 50 × 10^9^/L if bone marrow infiltration or splenomegaly ALT and AST level ≤ 3 x ULN Direct bilirubin level ≤ 2 x ULN, unless due to Gilbert’s syndrome CrCl ≥ 50mL/min (by Cockcroft-Gault formula) PT, INR, and aPTT ≤ 1.5 x ULN, unless receiving anticoagulation
Able to provide written informed consent
Women of childbearing potential (or their partners) must use at least one effective form of contraception plus a barrier method of contraception during trial participation
**Exclusion Criteria**
Current (or within 1 year) transformation to high grade lymphoma, including grade 3b follicular lymphoma (patients with historical high-grade transformation over 1 year ago are eligible)
Non-FDG avid disease
Prior allogenic stem cell transplantation (SCT) or solid organ transplant
Prior treatment with lenalidomide within 12 months of starting trial treatment
Treatment with CAR-T therapy within 100 days of starting trial treatment
SCT or maintenance therapy planned within 24 weeks of starting treatment (patients planning SCT/maintenance after at least 24 weeks of treatment are eligible)
Immunochemotherapy with a platinum-containing regimen planned
Known serological positivity for HIV or uncontrolled HCV
Hepatitis B surface antigen (HBsAg) positive and/or detectable viral DNA. Patients positive for Hepatitis B core antibody (anti-HBc) but viral DNA negative are eligible
Other malignancy within 2 years of enrolment, excepting cervical carcinoma stage 1B or less, non-invasive basal cell or squamous cell skin carcinoma, non-invasive, superficial bladder cancer, prostate cancer with a current PSA level < 0.1ng/mL, any curable cancer with a CR of > 2 years duration
Active systemic infection requiring treatment
Current or prior CNS involvement with lymphoma
History of allergy or anaphylaxis to anti-CD20 monoclonal antibody therapy
Known hypersensitivity to any of the novel arm IMPs. Patients with a known hypersensitivity to a control arm regimen may still be eligible if they have no hypersensitivity to other potential control arm IMPs.
Serious medical or psychiatric illness likely to interfere with participation in this clinical study
Recent cancer treatment (chemotherapy, immunotherapy, biological therapy) within 4 weeks of starting trial treatment; systemic steroid treatment (prednisolone > 10 mg daily (or equivalent)) within 7 days of cycle 1 day 1 dosing
Unwilling to use appropriate contraception methods whilst on study treatment and for 12 months following end of treatment (or 18 months for female patients whose ICT regimen contains obinutuzumab)
Women who are pregnant or breastfeeding
Prior treatment with a bispecific antibody
Major surgery within 30 days of starting treatment
Clinically significant cardiac disease including unstable angina within 6 months of study entry, acute MI within 6 months of study entry, New York Heart Association grade 3 or 4 congestive heart failure or known left ventricular ejection fraction < 45%)

ALT, Alanine transferase; ANC, Absolute neutrophil count; aPTT, Activated partial thromboplastin time; AST, Aspartate aminotransferase; CAR, Chimeric antigen receptor; CNS, Central nervous system; CR, Complete response; CrCl, Creatinine clearance; ECOG, Eastern cooperative oncology group; ICT, Investigator choice of standard therapy; IMP, Investigational medicinal product; INR, International normalised ratio; LVEF, Left ventricular ejection fraction; MUGA, Multi-gated acquisition; PET-CT, Positron emission tomography-computed tomography; PSA, Prostate-specific antigen; PT, Pro-thrombin; ULN, upper limit of normal

### Screening and consent

Potential patients will be identified at participating sites and multi-disciplinary team meetings. Exemplar informed consent forms and patient information sheets for the REFRACT trial are shown in Supplementary Appendices 3 and 4, respectively.

### Interventions

The REFRACT schedule of events for patients receiving ICT and epcoritamab + lenalidomide (R1) is shown in Supplementary Appendix 5.

The ICT arm includes an investigator choice of rituximab + bendamustine; rituximab + CVP; rituximab + CHOP; rituximab + lenalidomide; and obinutuzumab + bendamustine. Investigator choice treatments are delivered per local practice and protocols; suggested schedules are included in Supplementary Appendix 6. Patients randomised to ICT will receive treatment for up to twelve 28-day cycles (for rituximab + lenalidomide), up to six 28-day cycles (for rituximab or obinutuzumab + bendamustine), up to six 21-day cycles (for rituximab + CVP or CHOP), or until disease progression, unacceptable toxicity or patient choice, whichever comes first. At the investigator’s discretion, patients who respond to chemotherapy-based ICT may undergo autologous stem cell transplantation consolidation or receive single agent maintenance rituximab 375mg/m^2^ via intravenous (IV) or 1400 mg subcutaneous injection (or obinutuzumab 1000 mg IV for those who received obinutuzumab + bendamustine) once every two months for two years or until disease progression (whichever occurs first). These therapies are not considered investigational medicinal products or events.

Patients in R1 and randomised to epcoritamab + lenalidomide will receive treatment for up to twelve 28-day cycles or until disease progression, unacceptable toxicity, or patient choice, whichever comes first.

Lenalidomide 20 mg will be administered orally on days 1–21 of cycles 1–12 as per the summary of product characteristics.

Epcoritamab will be administered subcutaneously on days 1, 8, 15 and 22 of cycles 1 to 3 and on day 1 of cycles 4 to 12. Epcoritamab will be administered using a step-up dosing method as follows:

- Priming dose 0.16 mg on cycle 1 day 1.
- First intermediate dose 0.8 mg on cycle 1 day 8.
- Second intermediate dose 3 mg on cycle 1 day 15.
- Full dose 48 mg on cycle 1 day 22 and all subsequent dosing days.

Patient on epcoritamab will receive pre-medication with corticosteroids (mandatory during cycle 1, and in subsequent cycles if cytokine release syndrome (CRS) occurs), antihistamines, and antipyretics as described in Table 2. In addition, all patients treated with lenalidomide will receive mandatory prophylactic antithrombotic medicines per institutional standards.

**Table 2:** Pre-medication of patients receiving epcoritamab

			Corticosteroids	Antihistamines	Antipyretics
Cycle 1	1st epcoritamab administration (priming dose)	Day 1*	Dexamethasone 16 mg or Prednisolone 100 mg IV (or equivalent)	Chlorphenamine 10 mg PO/IV (or equivalent)	Paracetamol 1000 mg PO (or equivalent)
		Day 2	Dexamethasone 16 mg or Prednisolone 100 mg IV (or equivalent)		
		Day 3	Dexamethasone 16 mg or Prednisolone 100 mg IV (or equivalent)		
		Day 4	Dexamethasone 16 mg or Prednisolone 100 mg IV (or equivalent)		
	2nd epcoritamab administration (first intermediate dose)	Day 8*	Dexamethasone 16 mg or Prednisolone 100 mg IV (or equivalent)	Chlorphenamine 10 mg PO/IV (or equivalent)	Paracetamol 1000 mg PO (or equivalent)
		Day 9	Dexamethasone 16 mg or Prednisolone 100 mg IV (or equivalent)		
		Day 10	Dexamethasone 16 mg or Prednisolone 100 mg IV (or equivalent)		
		Day 11	Dexamethasone 16 mg or Prednisolone 100 mg IV (or equivalent)		
	3rd epcoritamab administration (second intermediate dose)	Day 15*	Dexamethasone 16 mg or Prednisolone 100 mg IV (or equivalent)	Chlorphenamine 10 mg IV (or equivalent)	Paracetamol 1000 mg PO (or equivalent)
		Day 16	Dexamethasone 16 mg or Prednisolone 100 mg IV (or equivalent)		
		Day 17	Dexamethasone 16 mg or Prednisolone 100 mg IV (or equivalent)		
		Day 18	Dexamethasone 16 mg or Prednisolone 100 mg IV (or equivalent)		
	4th epcoritamab administration (full dose)	Day 22*	Dexamethasone 16 mg or Prednisolone 100 mg IV (or equivalent)	Chlorphenamine 10 mg PO/IV (or equivalent)	Paracetamol 1000 mg PO (or equivalent)
		Day 23	Dexamethasone 16 mg or Prednisolone 100 mg IV (or equivalent)		
		Day 24	Dexamethasone 16 mg or Prednisolone 100 mg IV (or equivalent)		
		Day 25	Dexamethasone 16 mg or Prednisolone 100 mg IV (or equivalent)		
Cycle 2	5th epcoritamab administration (full dose)	Day 1*	If CRS > grade 1 occurs following the 4th epcoritamab administration, 4-day consecutive corticosteroid administration is continued in from Cycle 2 onwards as required	Optional	Optional

* 30 min to 2 h prior to administration of epcoritamab

CRS, Cytokine release syndrome; IV, Intravenous; PO, Oral administration

Granulocyte colony-stimulating factor and other hematopoietic growth factors may be used in the management of acute toxicity, such as febrile neutropenia and ≥ grade 3 neutropenia, when clinically indicated or at the investigator’s discretion. In case of recurring ≥ grade 3 neutropenia, use of growth factors is mandated

Patients will be followed up annually until the end of the trial. Quality of life questionnaires will be completed every 24 weeks in patients who have not progressed.

### Treatment compliance

The local trial pharmacist will be responsible for maintaining and updating drug accountability logs in the Pharmacy File for oral medications, which will be used to monitor compliance. At the end of treatment patients will be asked to return all unfinished bottles of medication to the site research team for reconciliation.

### Dose modifications and discontinuations

#### Cycle starting criteria

Lenalidomide treatment will not be started if the absolute neutrophil count is < 1 × 10^9^/L, or platelet count < 75 × 10^9^/L, or platelet count < 50 × 10^9^/L if due to bone marrow infiltration or splenomegaly. Treatment may be delayed by one week and abnormal results can be corrected at investigator’s discretion. Supportive care such as granulocyte colony stimulating factor (G-CSF) and transfusions may be given as required. If results are not corrected after one week dose reduction steps are required (shown in Table 3).

**Table 3:** Recommended dose modifications/delays for lenalidomide

Condition	Recommended Course of Action
**Thrombocytopenia**	
Platelets falls to < 50 × 10^9^/L	Interrupt lenalidomide treatment and conduct blood count at least every 7 days
Platelets return to ≥ 50 × 10^9^/L	Resume at next lower dose level (dose level − 1*)
For each subsequent drop below 50 × 10^9^/L	Interrupt lenalidomide treatment and conduct blood count at least every 7 days
For each subsequent return to ≥ 50 × 10^9^/L	Resume lenalidomide at next lower dose level (dose level − 2, -3*). Do not dose below dose level − 3.
**Neutropenia**	
ANC falls < 1.0 × 10^9^/L for at least 7 days or falls to < 1.0 × 10^9^/L with associated fever (body temperature ≥ 38.5 °C) or falls to < 0.5 × 10^9^/L	Interrupt lenalidomide treatment and conduct blood count at least every 7 days
ANC returns to ≥ 1.0 × 10^9^/L	Resume lenalidomide at next lower dose level (dose level − 1*)
For each subsequent drop below 1.0 × 10^9^/L for at least 7 days or drop to < 1.0 × 10^9^/L with associated fever (body temperature ≥ 38.5 °C) or drop to < 0.5 × 10^9^/L	Interrupt lenalidomide treatment and conduct blood count at least every 7 days
For each subsequent return to ≥ 1.0 × 10^9^/L	Resume lenalidomide at next lower dose level (dose level − 2, -3*). Do not dose below dose level − 3
**Renal Impairment**	
Moderate renal impairment (30 ≤ CrCl < 60 mL/min)	10 mg once daily^^^
Severe renal impairment (CrCl < 30 mL/min, not requiring dialysis)	At the discretion of the investigator
End stage renal disease (CrCl < 30 mL/min, requiring dialysis)	At the discretion of the investigator

ANC, absolute neutrophile count; CrCl, creatinine clearance

* Dose level − 1 = 15 mg once daily on days 1–21, every 28 days; dose level − 2 = 10 mg once daily on days 1–21, every 28 days; dose level − 3 = 5 mg once daily on days 1–21, every 28 days

^^^ At the physician’s discretion, if neutropenia is the only toxicity at any dose level, investigators may first support neutrophil count with granulocyte colony stimulating factor transfusions without reducing dose

### Lenalidomide

Dose modifications to lenalidomide can be applied at the investigator’s discretion, including in elderly, co-morbid, or heavily pre-treated patients. Recommended dose levels are shown in Table 3.

Patients experiencing grade 1 tumour lysis syndrome (TLS) may continue lenalidomide at the same dose or alternatively, at the investigator’s discretion, at a one dose level reduction. They should have intravenous hydration and appropriate medical management to correct abnormal laboratory values. In patients with grade 2 to 4 clinical TLS lenalidomide must be held. A chemistry panel should be performed as clinically indicated, and at least weekly. Vigorous intravenous hydration and appropriate medical management should be given according to the local standard of care until correction of electrolyte abnormalities. Lenalidomide may be restarted at the next lower dose level per investigator’s discretion when TLS resolves to grade 0.

Lenalidomide may be continued at the same dose in patients with grade 1 or 2 tumour flare reaction (TFR), without interruption or modification. At the investigator’s discretion, therapy with non-steroidal anti-inflammatory drugs (NSAIDs), a short course of corticosteroids, and/or opioid analgesics may be administered. In patients with grade 3 or 4 TFR, lenalidomide must be held and therapy with NSAIDs, corticosteroids and/or narcotic analgesics initiated. When TFR has resolved to grade ≤ 1, lenalidomide treatment may be restarted at the same dose level for the rest of the cycle.

For other grade 3 or 4 toxicities judged to be related to lenalidomide, treatment should be held and only restarted at next lower dose level when toxicity has resolved to grade ≤ 2 per investigator’s discretion. In addition, lenalidomide interruption or discontinuation should be considered for grade 2–3 skin rash. Lenalidomide must be permanently discontinued for angioedema, anaphylactic reaction, grade 4 rash, exfoliative or bullous rash, or if Stevens-Johnson syndrome, toxic epidermal necrolysis or drug reaction with eosinophilia and systemic symptoms is suspected.

### Epcoritamab

No dose reduction of epcoritamab is permitted in this trial. Epcoritamab may be delayed in the following circumstances:

- In the event a patient experiences a grade ≥ 3 adverse event (AE) considered related to epcoritamab, the epcoritamab dose should be held until the AE resolves to grade ≤ 1 (or, for a pre-existing condition, to baseline severity). If the scheduled dose is delayed beyond four weeks, continuation of epcoritamab treatment should be discussed with a Clinical Coordinator.
- If the interval between epcoritamab doses exceeds six weeks, a Clinical Coordinator should be contacted to discuss continuing epcoritamab. When re-starting epcoritamab after a delay, four days of corticosteroids may be considered, until at least one full dose is administered without subsequent occurrence of CRS grade ≥ 2.
- A re-priming cycle of epcoritamab must be administered in the following situations:
- Cycle 1 day 8 is delayed more than one day (i.e., cycle 1 day 8 is planned to occur more than eight days after cycle 1 day 1).
-
  - Cycle 1 day 15 is delayed more than one day (i.e., cycle 1 day 15 is planned to occur more than 8 days after priming or any intermediate dose).
  - Cycle 1 day 22 is delayed more than seven days (i.e., more than 14 days since the last intermediate dose).
  - For cycle 2 day 1 onward, if the interval between the previous dose of epcoritamab and next planned dose exceeds six weeks.
- Epcoritamab re-priming will be administered using a step-up dosing method as follows:
-
  - Priming dose 0.16 mg on first day.
  - Intermediate dose 0.8 mg 7 days later.
  - Second intermediate dose 3 mg 7 days later.
  - Full dose 48 mg 7 days later and all subsequent dosing days (patient to return to protocol schedule, i.e., weekly if on cycle 1 to 3, 4-weekly thereafter).

Pre-medication with corticosteroids, antihistamines and antipyretics is mandated during epcoritamab re-priming.

Lenalidomide dosing may resume/continue during epcoritamab re-priming if there are no criteria for lenalidomide dose reduction or delays.

- In cases of grade ≥ 4 cytokine release syndrome, epcoritamab must be permanently discontinued.
- In the event a patient experiences a second episode of the same AE at grade ≥ 3 which is considered related to epcoritamab, continuation of epcoritamab must be discussed with a Clinical Coordinator.

If either epcoritamab or lenalidomide is discontinued, the other drug may be continued as monotherapy only following discussion with the Clinical Coordinator.

### Investigator choice of standard therapy

Standard dose modifications to ICT apply as per local practice or at the investigator’s discretion. Recommendations are included in Supplementary Appendix 7.

### Additional supportive treatment

Anti-emetic prophylaxis should be provided to all patients receiving ICT, including gastric protection for those receiving high-dose steroids and bone protection per local protocols. In addition, infusion-related reactions are common after administration of rituximab and obinutuzumab, therefore, pre-medication is mandatory (see Supplementary Appendix 8).

Prophylaxis against herpes virus infection/reactivation and *Pneumocystis jirovecii* infection is strongly recommended. Hepatitis B reactivation must be monitored and treated according to local protocols. Primary prophylaxis against neutropenia with G-CSF should be considered particularly in patients at higher risk of infection. Prophylaxis against TLS should consist of adequate hydration and administration of uricostatics or a suitable alternative treatment such as urate oxidase.

### Concomitant medication

The use of live vaccines is not recommended in any treatment arm, unless unavoidable.

In patients receiving lenalidomide, co-administration with digoxin may increase the plasma exposure of digoxin; digoxin monitoring is advised. Co-administration with statins increased the risk of rhabdomyolysis; enhanced monitoring is recommended during the first weeks of treatment.

There are no concomitant medication restrictions for epcoritamab.

Supplementary Appendix 9 has information on concomitant medication for patients receiving ICT.

### Trial outcomes

The primary outcome of the trial is complete metabolic response (CMR) by positron emission tomography-computed tomography (PET-CT) at 24 weeks from the start of induction therapy using the Deauville 5-point scale and Lugano 2014 criteria [15]. PET is more sensitive than CT for response assessment in FL and a powerful predictor of long-term disease control and survival [16–20].

Secondary outcomes include:

- Overall metabolic response (OMR; CMR + partial metabolic response (PMR)) by PET-CT at 24 weeks.
- Progression free survival (PFS) defined as the time from randomisation to the date of first disease progression or death from any cause.
- Overall survival defined as time from randomisation to the date of death from any cause.
- Duration of response (DoR) defined as the time from complete and partial metabolic response by PET-CT to relapse/progression or death from any cause.
- Duration of complete response (DoCR) defined as the time from complete metabolic response by PET-CT to relapse/progression or death from any cause.
- Time to next treatment (TTNT) defined as the time from randomisation to the start date of next treatment for lymphoma. For patients who are responding (CMR or PMR) and receive consolidation radiotherapy, radiotherapy will not be considered an event. Patients will be censored at their date last seen if no other treatment for disease related reasons is reported. Patients who die without having started next lymphoma treatment will be censored at their date of death, and patients who are alive at the end of the trial and have not started next lymphoma treatment will be censored at their date last seen.
- Adverse events (AEs) collected and reported in accordance with CTCAE version 5.0 [21] defined as the number of patients who experience one or more grade 3 or 4 AEs or serious AEs (SAEs) of any grade.
- Quality of life (measured using the EQ-5D-5 L [22] and Functional Assessment of Cancer Treatment–Lymphoma (FACT-Lym) [23]) collected pre-treatment, day 1 of cycle 3 and at 24 weeks from treatment start and then every 24 weeks in non-progressed patients until the end of study.

Quality of life questionnaires, EQ-5D-5 L [22] (standardised instrument for measuring generic health status) and FACT-Lym [23] (a validation measure of QoL for non-Hodgkin’s lymphoma patients) will be completed independently by patients.

Exploratory outcome measures include:

- Exploring the prognostic value of PET-CT radiomic features including PET-CT total metabolic tumour volume.
- Characterising and evaluating the predictive value of dynamic changes in the tumour microenvironment (TME) using deconvoluted bulk cell RNA sequencing.
- Evaluating the predictive and prognostic value of baseline, interim and end of treatment circulating tumour (ct)DNA levels and the peripheral blood immune composition.
- Classifying TME classes using imaging mass cytometry, identifying predictive and treatment guiding biomarkers and new druggable targets.

### Statistical analysis plan

Efficacy analyses will be conducted on a modified intention-to-treat (mITT) population, including any patient that discontinues treatment or is found to be ineligible post randomisation where ineligibility is not deemed to impact patients’ response to treatment. Patients will be replaced if they undergo stem-cell transplant (SCT) within 24 weeks of randomisation, fail to start treatment, or if ineligibility is deemed to impact upon response to treatment. Patients who are replaced will not be included in the mITT population. A safety population will include all patients who were eligible for the trial and who started trial treatment.

The primary outcome of complete metabolic response by PET-CT at 24 weeks will be reported as number and proportions, with the numerator being the number of patients achieving a complete response and the denominator being the total number of patients within the mITT population. Bayesian posterior probability plots will be presented alongside the probability that the true difference between the treatment arms surpasses a range of relevant thresholds (10%, 15% & 20%). For R1 a minimally informative beta prior of Beta [1] will be employed, in future rounds this prior will be informed by previous control round response rates and both the prior and weightings utilised within these analyses will be reported. Success, here being determination that a treatment should be investigated further, is defined as finding a greater than 60% probability that the true difference between the novel and control arm is greater than 15%: *Prob(true difference between arms ≥ 15%) ≥ 60%.*

A detailed secondary outcome measure analysis plan can be found in the predefined statistical analysis plan (Supplementary Appendix 10). The trial statisticians will not be blinded. Exploratory subgroup analyses will be conducted to ascertain the effect of treatment on the primary outcome measure within disease risk score (high risk vs. not high risk).

### Sample size determination

Based on feasibility assessments a total sample size for all three rounds of 284 patients (95 control + 189 novel arm patients in total) was deemed appropriate. R1 will treat 126 patients (63 patients per arm, 1:1 randomisation). R2 and R3 will treat 63 patients in the novel arm and 16 in the control arm (4:1 randomisation). In order to make the most efficient use of patients’ contributions and reduce the number of control arm patients required in R2 and 3, data from patients recruited to previous control arms will be incorporated into subsequent rounds using power priors [24]. Bayesian operating characteristics were used to calculate the probability that the PET-CT CMR rate in the novel arm is greater than a given value, under predefined conditions. A more detailed description of the Bayesian methodology using power priors is described elsewhere (manuscript submitted), sample size determinations can be found in the predefined statistical analysis plan (Supplementary Appendix 10).

### Positron emission tomography sub-study

The prognostic role of tumour burden assessed by measurement of metabolic tumour volume (MTV) has been reported in retrospective analysis of clinical trials in patients receiving first line treatment [25]. The role of baseline MTV has not been explored prospectively in the rrFL setting or when using chemotherapy-free regimens. A planned PET sub-study will determine whether baseline MTV is prognostic and useful for risk stratification, and whether emerging radiomic features in tumour and uninvolved ‘healthy’ tissues in combination with host related factors may provide additional prognostic information [26, 27]. This study will also explore the relationship between PET findings and non-imaging biomarkers from other translational sub-studies, for example tumour microenvironment and mutational profiling at relapse, clearance of ctDNA at end of induction and whether intra-patient heterogeneity on imaging can identify sites with differing mutational analysis.

### Other translational sub-studies and associated sample collection

The composition of TME is prognostic in FL [28] and may make a critical contribution to response and resistance mechanisms during therapy. Tumour formalin-fixed paraffin-embedded tissue samples collected at initial FL diagnosis, screening and subsequent relapse/progression will support studies to characterise the tumour extrinsic tumour microenvironment. Specifically, the pre-treatment composition and spatial distribution of immune and stromal/fibroblast cells will be characterised using high dimensional imaging mass cytometry and equivalent technologies for correlation with treatment outcomes to identify novel biomarkers and new druggable targets. Single cell and bulk RNAseq will further define dynamic changes underpinning treatment response, resistance to therapy, and subsequent disease relapse.

Peripheral blood samples and bone marrow aspirates collected at screening, during and after treatment, and at subsequent relapse/progression will support studies examining the tumour extrinsic peripheral blood cellular immunome as a predictor of therapeutic response as well as investigating ctDNA as a tumour intrinsic dynamic response surveillance tool in rrFL.

Saliva will be collected during screening for germline DNA analysis.

All samples will be collected in accordance with national regulations and requirements including standard operating procedures for logistics and infrastructure. Samples will be taken in appropriately licensed premises and transported in accordance with the Human Tissue Authority guidelines and NHS trust policies.

### Adverse events reporting and analysis

The collection and reporting of AEs as measured by National Cancer Institute (NCI) Common Terminology Criteria for Adverse Events (CTCAE), version 5.0 [21], will be in accordance with the Research Governance Framework for Health and Social Care and the requirements of the National Research Ethics Service. Definitions of different types of AEs are listed in online Supplementary Appendix 11. The reporting period for AEs will be documented and reported from the date of commencement of protocol defined treatment until 60 days after the administration of the last dose of protocol treatment in those patients within the novel arms, and until 12 months + 60 days for patients receiving ICT.

The investigator should assess the seriousness and causality (relatedness) of all AEs experienced by the patient (this should be documented in the source data) with reference to the protocol. All medical occurrences which meet the definition of an AE must be reported for patients randomised to a novel arm treatment. All grade 3 and above medical occurrences which meet the definition of an AE should be reported for those receiving ICT, with the exception of abnormal laboratory findings which should only be reported if the event results in the early discontinuation of trial treatment and/or requires a dose modification or interruption or any other therapeutic intervention or is judged to be of significant clinical importance.

If a laboratory abnormality is one component of a diagnosis or syndrome, then only the diagnosis or syndrome will be recorded as an AE. Pre-existing conditions will only be reported if the condition worsens by at least one CTCAE grade.

Hospitalisations for protocol defined treatment, pre-planned elective procedures without worsening of the condition, treatment for lymphoma progression and progression or death as a result of lymphoma will not be reported as SAEs as this information is captured elsewhere on the Case Report Form.

An Adverse Event of Special Interest (AESI) is one of scientific and medical interest specific to understanding of the protocolised drug and may require close monitoring. An AESI may be serious or non-serious. The following AESIs may be associated with epcoritamab and should be reported as a SAE: cytokine release syndrome of any grade; immune effector cell-associated neurotoxicity syndrome of any grade; any suspected hemophagocytic lymphohistiocytosis of any grade; clinical TLS; neutropenic sepsis.

### Data management

Case report forms (CRF) are entered into a secure online database. Authorised staff at sites will require an individual secure login username and password to access this online data entry system. For the purposes of this trial the QoL questionnaires will be captured on paper and entered onto the eRDC system by the REFRACT Trial Office. Data reported on each CRF should be consistent with the source data or the discrepancies should be explained. If information is not known, this must be clearly indicated on the CRF. All missing and ambiguous data will be queried. All sections are to be completed.

All trial records must be archived and securely retained for at least 25 years. No documents will be destroyed without prior approval from the sponsor, via the central REFRACT Trial Office. On-site monitoring will be carried out as required following a risk assessment and as documented in the Quality Management Plan. Any monitoring activities will be reported to the central REFRACT Trial Office and any issues noted will be followed up to resolution. REFRACT will also be centrally monitored, which may trigger additional on-site monitoring.

The Cancer Research UK Clinical Trials Unit (CRCTU) will hold the final trial dataset and will be responsible for the controlled sharing of anonymised clinical trial data with the wider research community to maximise potential patient benefit while protecting the privacy and confidentiality of trial participants. Data anonymised in compliance with the Information Commissioners Office requirements, using a procedure based on guidelines from the Medical Research Council Methodology Hubs, will be available for sharing with researchers outside of the trials team within 12 months of the primary publication.

### Trial organisation structure

The University of Birmingham will act as single sponsor for this multi-centre study: Support Group, Aston Webb Building, Room 119, Birmingham, B15 2TT. Email: researchgovernance@contacts.bham.ac.uk. The trial is conducted under the auspices of the CRCTU, University of Birmingham according to their local procedures. The TMG will be responsible for the day-to-day running and management of the trial. Members of the TMG include the Chief Investigators, University of Birmingham lead investigator, co-investigators, patient representatives, sub-study specialists, the trial management team leader (or delegate), the trial biostatistician, trial coordinator, and monitor. The TMG will have regular meetings during recruitment.

An independent TSC will be set up to oversee the conduct of the trial. The TSC will be led by the independent Chair, with membership including an independent clinician, independent statistician, a patient advocate, and a representative from the sponsor. Selected members of the TMG including the Chief Investigators, and the trial biostatistician and co-investigators will report to the TSC. The TSC will operate in accordance with a trial specific charter based upon the template created by the Damocles Group to supervise the conduct of the trial, monitoring progress including recruitment, data completeness, losses to follow-up, and deviations from the protocol. They will make recommendations about conduct and continuation of the trial to the sponsor. The TSC will meet shortly before commencement of the trial and then 6-monthly thereafter after the data management committee (DMC) meeting.

The DMC will consist of independent clinicians, as well as an independent statistician. Data analyses will be supplied in confidence to the DMC, which will be asked to give advice on whether the accumulated data from the trial, together with the results from other relevant research, justifies the continuing recruitment of further patients. The DMC will meet at least annually while patients are on treatment. Additional meetings may be called if recruitment is much faster than anticipated and the DMC may, at their discretion, request to meet more frequently. An emergency meeting may also be convened if a safety issue is identified. The DMC will report to the TMG who will convey the findings of the DMC to the TSC and the UK’s competent authority the Medicines and Healthcare Products Regulatory Agency (MHRA). The DMC may consider recommending the discontinuation of the trial if the recruitment rate or data quality are unacceptable or if any issues are identified which may compromise patient safety.

### Confidentiality

Confidential information collected during the trial will be stored in accordance with the General Data Protection Regulation (GDPR) 2018. As specified in the PIS and with the patients’ consent, patients will be identified using only their date of birth and unique trial ID number. Authorised staff may have access to the records for quality assurance and audit purposes. The Trials Office maintains the confidentiality of all patients’ data and will not disclose information by which patients may be identified to any third party other than those directly involved in the treatment of the patient and organisations for which the patient has given explicit consent for data transfer (e.g., laboratory staff).

### Dissemination of results and publication policy

A meeting will be held after the end of the study to discuss main results with collaborators prior to publication. Results of the primary and secondary endpoints will be submitted for publication in peer-reviewed journals. Manuscripts will be prepared by the TMG, and authorship determined by mutual agreement. A lay summary of the results will be published on the Cancer Research UK website.

### Trial status

Recruitment for the trial opened on 13-Jul-2023 and recruitment is expected to last five years.

## Discussion

### Trial design

REFRACT is a randomised platform study designed to permit an unbiased comparison of treatment arms and to avoid well-recognised confounding factors inherent to non-randomised comparisons, allowing evidence of therapeutic efficacy to be established. Randomisation for round 1 (R1) will utilise a 1:1 allocation ratio to either novel or standard control treatment. Following R1, subsequent rounds will utilise a 1:4 randomisation allocation in favour of the novel arm and employ a Bayesian data sharing strategy in order to provide adequate data for the control arm. This randomisation procedure has been chosen as the control arm is unlikely to change over the course of the trial and, as such, data from earlier-recruited control patients can contribute to the analysis of later rounds. The implementation of Bayesian power prior methodology within the platform setting allows for the incorporation of control data into later rounds, increasing the effective sample size as well as allowing for adaptive weighting of control data determined according to the similarity of control arms over the lifetime of the trial. The use of PET CMR as the primary outcome reduces the time to data maturity, meaning that treatments with an efficacy signal will be identified and reported in a relatively short time frame. This also allows investigators to offer post-induction options such as rituximab maintenance and autologous stem cell transplantation without affecting the trial primary endpoint. Moreover, prospective efficacy and safety data collected from patients on the control arm will provide a new benchmark to inform the ongoing design of this and future clinical trials, and an evidence base to inform regulatory approval of novel therapies.

This highly efficient design minimises patient numbers required to investigate three novel therapies and accelerates trial delivery in a rapidly evolving disease landscape, therefore maximising the overall efficiency of the trial. An embedded cross-cutting programme of translation research will elucidate the biological determinants of treatment response and resistance, identify sensitive subsets, and derive companion diagnostics and biomarkers for patient selection, especially in high-risk and multiple relapsed settings.

### Patient perspective

There are many people in the UK who have been diagnosed with Follicular Lymphoma, and who have been treated successfully. They know that the disease may come back and they may need further treatment. Fortunately many new, more potent treatments for lymphoma have been developed in the last few years, and the REFRACT study is designed to provide the evidence that clinicians will need to decide which treatment to prescribe. The same evidence will be needed to help NICE to decide if the new medicine should be made available to patients on the NHS. If epcoritamab with lenalidomide proves successful, the trial participants receiving it will have better outcomes than they would have done outside the trial, and future patients will have better outcomes if it is adopted by the NHS. REFRACT could therefore accelerate access to a substantially better treatment, allowing people to live longer and with better quality of life than previously, after their disease come back.

### Electronic supplementary material

Below is the link to the electronic supplementary material.

Supplementary Material 1

Supplementary Material 2

Supplementary Material 3

Supplementary Material 4

Supplementary Material 5

Supplementary Material 6

Supplementary Material 7

Supplementary Material 8

Supplementary Material 9

Supplementary Material 10

Supplementary Material 11

Supplementary Material 12

## Data Availability

No datasets were generated or analysed during the current study.

## Declarations

All patients provide written informed consent to participate.

## Abbreviations

AE: Adverse event
AESI: Adverse event of special interest
ANC: Absolute neutrophile count
CMR: Complete metabolic response
CR: Complete response
CrCl: Creatinine clearance
CTCAE: Common terminology criteria for adverse events
CYP: Cytochrome P450
DMC: Data management committee
DoCR: Duration of complete response
DoR: Duration of response
FACT-Lym: Functional Assessment of Cancer Treatment–Lymphoma
FDG: Fluorodeoxyglucose
FL: Follicular lymphoma
G-CSF: Granulocyte colony stimulating factor
ICT: Investigator choice of standard therapy
MHRA: Medicines and Healthcare Products Regulatory Agency
NCRI: National Cancer Research Institute
NICE: National Institute for Health and Care Excellence
OS: Overall survival rate
OMR: Overall metabolic response
ORR: Overall response rate
PET-CT: Positron emission tomography-computed tomography
PFS: Progression free survival rate
PMR: Partial metabolic response
QoL: Quality of life
R: Round
REFRACT: Relapsed Follicular lymphoma Randomised trial Against standard ChemoTherapy
rrFL: Relapsed or refractory follicular lymphoma
SAE: Serious adverse event
TLS: Tumour lysis syndrome
TME: Tumour microenvironment
TMG: Trial management group
TSC: Trial steering committee
TTNT: Time to next treatment
