# Supplementary material for: Investigator choice of standard therapy versus sequential novel therapy arms in the treatment of relapsed follicular lymphoma (REFRACT): study protocol for a multi-centre, open-label, randomised, phase II platform trial
Source: BMC Cancer. 2024 Mar 25;24:370. doi: 10.1186/s12885-024-12112-0 (PMC10962099; doi:10.1186/s12885-024-12112-0)
Supplement: Supplementary file 1 — Supplementary Material 1 [file 12885_2024_12112_MOESM1_ESM.docx]

**Additional Files**

**Supplementary Appendix 1: SPIRIT checklist for the REFRACT protocol**

A completed Standard Protocol Items: Recommendations for Intervention Trials checklist for the REFRACT protocol.

**Supplementary Appendix 2:** **WHO trial registration data set for the REFRACT trial**

The World Health Organization trial registration data set for the REFRACT trial.

**Supplementary Appendix 3: REFRACT informed consent forms**

Exemplar informed consent and blood sample analysis consent form for the REFRACT trial.

**Supplementary Appendix 4: REFRACT patient information sheets**

Exemplar trial and blood sample analysis patient information sheets for REFRACT.

**Supplementary Appendix 5: REFRACT schedule of events**

Patient schedule of events for the REFRACT trial.

**Supplementary Appendix 6: Recommended treatment schedules for the permitted investigator choice of standard therapies**

A list of recommended schedules for therapies permitted within the investigator choice of standard therapy (ICT) arm of the REFRACT trial.

**Supplementary Appendix 7: Recommended dose modifications for the permitted investigator choice of standard therapies**

A list of recommended dose modifications for therapies permitted within the investigator choice of standard therapy (ICT) arm of the REFRACT trial.

**Supplementary Appendix 8: Pre-medication to prevent infusion-related reactions**

Treatment schedules to prevent infusion-related reactions in patients receiving rituximab or obinutuzumab in the REFRACT trial.

**Supplementary Appendix 9: Concomitant medications for patients receiving investigator choice of standard therapy**

A list of those medications that should be used with caution in patients receiving investigator choice of standard therapy (ICT) in the REFRACT trial.

**Supplementary Appendix 10: Statistical analysis plan**

Pre-defined plan for statistical analyses

**Supplementary Appendix 11: Adverse event definitions**

Definitions of adverse events used for the REFRACT trial.
