# Supplementary material for: Investigator choice of standard therapy versus sequential novel therapy arms in the treatment of relapsed follicular lymphoma (REFRACT): study protocol for a multi-centre, open-label, randomised, phase II platform trial
Source: BMC Cancer. 2024 Mar 25;24:370. doi: 10.1186/s12885-024-12112-0 (PMC10962099; doi:10.1186/s12885-024-12112-0)
Supplement: Supplementary file 3 — Supplementary Material 3 [file 12885_2024_12112_MOESM3_ESM.docx]

# Supplementary Appendix 2: WHO trial registration data set for the REFRACT trial

| **Data category** | **Information** |
| --- | --- |
| Primary registry and trial identifying number | EudraCT Number 2022-000677-75 |
| Date of registration in primary registry | 10 February 2022 |
| Secondary identifying numbers | ClinicalTrials.gov NCT05848765 08 May 2023 |
| Source(s) of monetary or material support | Cancer Research UK (CRCCTA-Dec21\100004).  Genmab |
| Primary sponsor | University of Birmingham |
| Secondary sponsor(s) | n/a |
| Contact for public queries | REFRACT trial office: [REFRACT@trials.bham.ac.uk](mailto:REFRACT@trials.bham.ac.uk) |
| Contact for scientific queries | REFRACT trial office: [REFRACT@trials.bham.ac.uk](mailto:REFRACT@trials.bham.ac.uk) |
| Public title | n/a |
| Scientific title | Relapsed Follicular lymphoma Randomised trial Against standard ChemoTherapy (REFRACT): A randomised phase II trial of investigator choice standard therapy versus sequential novel therapy experimental arms |
| Countries of recruitment | UK |
| Health condition(s) or problem(s) studied | Relapsed or refractory follicular lymphoma; |
| Intervention(s) | Control: Investigator’s choice of standard therapy (ICT). Permitted regimens are:  Rituximab and bendamustine; rituximab and CVP; rituximab and CHOP; rituximab and lenalidomide; and obinutuzumab and bendamustine.  Experimental: Round 1 = Epcoritamab + lenalidomide  Experimental treatments for Rounds 2 and 3 are yet to be determined |
| Key inclusion and exclusion criteria: | Ages eligible for study: Aged 18 years and over Sexes eligible for study: Both Accepts healthy volunteers: No |
|  | Inclusion criteria: Biopsy proven relapsed or refractory CD20 positive, grade 1-3a follicular lymphoma, performance status <2, assessable disease by PET-CT (at least one involved node with long diameter >1.5cm, or extranodal lesion >1cm) |
|  | Exclusion criteria: Prior allogenic stem cell transplantation or solid organ transplant, pregnant or breastfeeding patients |
| Study type | Interventional |
|  | Allocation: Randomised, open-label |
|  | Primary purpose: Efficacy |
|  | Phase II |
| Date of first enrolment | 04 September 2023 |
| Target sample size | 284: 95 control and 189 experimental arm patients |
| Recruitment status | Open |
| Primary outcome(s) | Complete metabolic response (CMR) by PET-CT at 24 weeks from the start of induction therapy using the Deauville 5-point scale and Lugano 2014 criteria |
| Key secondary outcome(s) | • Overall metabolic response (OMR; CMR + partial metabolic response (PMR)) by PET-CT at 24 weeks.  • Progression free survival (PFS) defined as the time from randomisation to the date of first disease progression or death from any cause.  • Overall survival defined as time from randomisation to the date of death from any cause.  • Duration of response (DoR) defined as the time from complete and partial metabolic response by PET-CT to relapse/progression or death from any cause  • Quality of life (measured using the EQ-5D-5L and FACT-Lym) collected pre-treatment, day 1 of cycle 3 and at 24 weeks from treatment start and then every 24 weeks in non-progressed patients until the end of study |
