# Supplementary material for: Investigator choice of standard therapy versus sequential novel therapy arms in the treatment of relapsed follicular lymphoma (REFRACT): study protocol for a multi-centre, open-label, randomised, phase II platform trial
Source: BMC Cancer. 2024 Mar 25;24:370. doi: 10.1186/s12885-024-12112-0 (PMC10962099; doi:10.1186/s12885-024-12112-0)
Supplement: Supplementary file 4 — Supplementary Material 4 [file 12885_2024_12112_MOESM4_ESM.docx]

### Supplementary Appendix 3: REFRACT informed consent forms


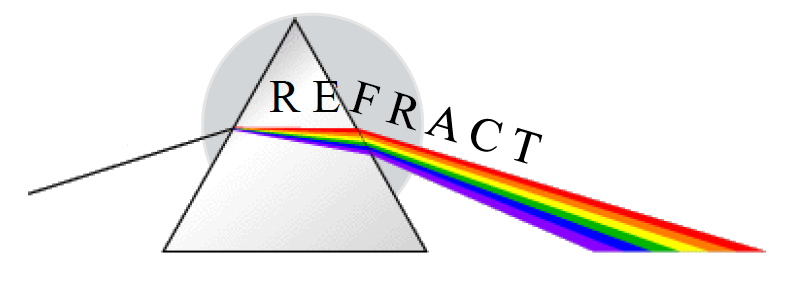


Informed Consent Form

**Round 1 – Epcoritamab + Lenalidomide**

Version 1.0 6^th^ October 2022

**REFRACT: A randomised phase II trial of investigator choice standard therapy versus sequential novel therapy experimental arms in relapsed and refractory follicular lymphoma**

| Site: |  | Patient’s Trial  Number: |  |
| --- | --- | --- | --- |
| Principal Investigator: |  | **EudraCT No.:** 2022-000677-75 | |

### Please initial each box

| 1. **Taking part** 2. I confirm that I have read and understood the Patient Information Sheet (version .................... dated...........................) for Round….. of the REFRACT trial. I have had the opportunity to consider the information, ask questions and have had these answered satisfactorily. | | |  |
| --- | --- | --- | --- |
| 1. I understand that my participation is voluntary and that I am free to withdraw at any time without giving a reason, without my medical care or legal rights being affected. I understand that if I withdraw, my data and samples collected up until the point of withdrawal will still be analysed. | | |  |
| 1. I give my permission for my date of birth to be given to the Trial Office when I am randomised to the REFRACT trial and for a copy of this consent form, which will not be anonymised, to be given to the Trial Office and Liverpool Biobank. 2. I agree to my GP being informed of my participation in this trial. 3. I agree to take part in the REFRACT trial. | | |  |
| 1. **Research samples** 2. I agree to donate samples of my tumour biopsies (including the tumour tissue collected within the last 3 months or at screening, and any subsequent biopsies I have during the trial), saliva, blood and bone marrow as part of participation in the REFRACT study. | | |  |
| 1. I understand that my samples will be used for research studies associated with this trial as described in the Patient Information Sheet. | | |  |
| 1. I agree to allow samples to be supplied to the Liverpool Biobank, as outlined in the Patient Information Sheet, for storage, and sent to the University of Cambridge, Barts Cancer Institute and Kings College London as part of the analysis for this study (including the extraction, analysis and storage of my DNA). 2. I give consent for the data from my PET and PET-CT scans to be sent to approved PET facilities at Kings College London and used for research associated with this trial. | | |  |
| **Optional -** The following is optional and will not affect entry into the trial, please initial for no or yes in the boxes:   1. I agree to the storage of my samples remaining at the end of the study and their use in existing or future research to better understand relapsed/refractory follicular lymphoma and to help develop new treatments for the disease. This research may involve genetic analysis. The results of any genetic tests will not affect my medical care. I understand that my samples or DNA from my samples could be shared with any UK or overseas research organisation. Research organisations may include academic institutions, clinical research groups or commercial (for-profit) companies. Any existing or future research carried out on my samples will have the relevant country specific approvals (including ethical approval). I understand that future research may use new tests or techniques that are not yet known. 2. I give consent for tissue from my tumour biopsy at my initial diagnosis of Follicular Lymphoma to be sent to the Liverpool Biobank and used for research associated with this trial. | | | **No Yes** |
| 1. **Data** 2. I understand that relevant sections of my medical notes and data collected during the study may be looked at by individuals from the Trial Office, regulatory authorities, drug manufacturers, Sponsors, research collaborators, and/or NHS bodies, where it is relevant to my taking part in this research, safety monitoring, or licencing purposes. I give permission for these individuals to have access to my records. | | |  |
| 1. I understand that my data, and information from my samples will only be used by researchers in a form that protects my anonymity. Anonymised data and information derived from research samples may be shared with other research organisations in future research. This may include academic institutions, clinical research groups or commercial (for-profit) companies. I understand that this data may be transmitted outside the European Economic area to countries which may have a different level of data protection to that in the UK. | | |  |
|  | | |  |
| **____________________________** | **_____________** | **_______________________________** | |
| **Name of participant** | **Date** | **Signature** | |
|  |  |  | |
| **____________________________** | **_____________** | **_______________________________** | |
| **Name of person taking consent**  (You must have signed the Site Signature and Delegation Log) | **Date** | **Signature** | |
|  |  |  | |
| When completed, 1 for patient; 1 (original) for Investigator Site File; 1 to be kept in medical notes; 1 to be sent to CRCTU and 1 to be sent to the Liverpool Biobank. | | | |
