# Supplementary material for: Investigator choice of standard therapy versus sequential novel therapy arms in the treatment of relapsed follicular lymphoma (REFRACT): study protocol for a multi-centre, open-label, randomised, phase II platform trial
Source: BMC Cancer. 2024 Mar 25;24:370. doi: 10.1186/s12885-024-12112-0 (PMC10962099; doi:10.1186/s12885-024-12112-0)
Supplement: Supplementary file 5 — Supplementary Material 5 [file 12885_2024_12112_MOESM5_ESM.docx]

**Supplementary Appendix 4: REFRACT patient information sheet**


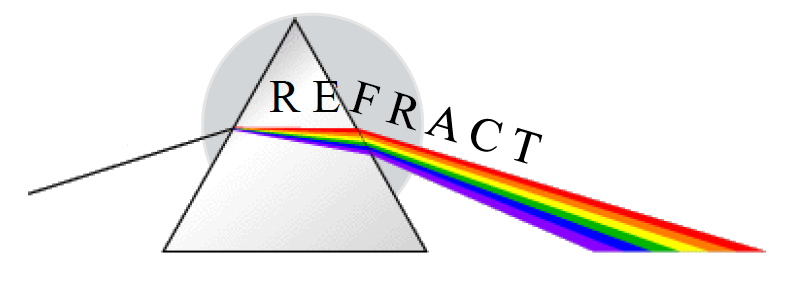


**Patient Information Sheet**

**Round 1 – Epcoritamab + Lenalidomide**

Version 3.0 17^th^ May 2023

**REFRACT: A randomised phase II trial of investigator choice standard therapy versus sequential novel therapy experimental arms in relapsed and refractory follicular lymphoma**

| We would like to invite you to take part in a research study (also called a clinical trial) which is sponsored by the University of Birmingham. Joining the study is entirely up to you; before you decide we would like you to understand why the research is being done and what it would involve for you. Please take time to read this information carefully and discuss it with others if you wish.  **Part 1** tells you the purpose of this study and what will happen to you if you take part.  **Part 2** gives you more detailed information about the conduct of the study.  Please ask if anything is unclear, or if you would like more information. Take time to decide whether or not you wish to take part. If you choose not to take part, this will not affect the care you receive from your own doctors.  Thank you for reading this information sheet. |
| --- |

| **Contents**  **Part 1**  What is the purpose of the REFRACT study?  Why have I been invited to take part?  How do I enter the study?  Do I have to take part?  What will happen if I decide to take part?  What samples will be taken?  What will happen to the samples at the end of the study?  What are the possible benefits of taking part?  What are the possible disadvantages and risks  of taking part? | **Page**  3  3  4  4  4  14  15  15  16 | **Contents**  **Part 2**  Will I be paid to take part?  What if there are problems?  What if new information becomes available?  Will my taking part in the study be kept confidential?  Will any genetic tests be done?  What will happen to the results of the study?  Who is organising and funding the study?  Who has reviewed the study?  How have patients been involved?  Further information and contact details | **Page**  23  23  23  23  25  26  26  26  26  27 |
| --- | --- | --- | --- |

**Trial Summary**

The REFRACT study will compare new and current treatments for Follicular Lymphoma patients who have had their cancer return after treatment (relapsed) or did not respond well to treatment (refractory).

You are being invited to join round 1, which is a study of epcoritamab combined with lenalidomide. Epcoritamab is a new treatment that is likely to work at least as well as current treatments (based on previous studies). We hope it may increase the time before additional treatment is necessary. It may have other benefits as well such as fewer side-effects and more convenient delivery. The aim of the study is to see if these early hopes are correct or not. Lenalidomide is a drug that is currently used in the treatment of follicular lymphoma.

If you decide to take part in the study you will receive either epcoritamab + lenalidomide or a standard treatment (chosen by your doctor). There is an equal chance of receiving the standard or the new treatment and this will be decided at random by a computer.

If you decide not to take part, you will be offered the same standard treatment. Your doctor will explain which of the standard treatments they recommend for you.

No-one will receive a placebo or a treatment that is not recommended by their doctor.

If your lymphoma returns during the study, or does not respond to the treatment, you will be offered a different treatment by your doctor and you will stop receiving study treatment.

Future patients will benefit from the results of this study as doctors will have better evidence on which treatments to offer different patients. Some patients may benefit from fewer side-effects, others may stay in remission for longer. Epcoritamab is not yet available on the NHS and this study may speed up the process of deciding if it should be made available or not, and which patients should receive it.

| **Trial Treatment Options** | **Number of Hospital visits for treatment** | **Length of treatment** | **Number of Additional Procedures for study participants** |
| --- | --- | --- | --- |
| **Standard** |  |  | PET-CT scan pre-treatment and saliva sample for research |
| Rituximab + CHOP* **OR** | 6 | 18 weeks |  |
| Rituximab + CVP* **OR** | 6 | 18 weeks |  |
| Rituximab + Bendamustine* **OR** | 15 | 24 weeks |  |
| Obinutuzumab + Bendamustine* **OR** | 14 | 24 weeks |  |
| Rituximab + lenalidomide | 12 | 48 weeks |  |
| *With these treatments rituximab or obinutuzumab maintenance may be received every 2 months for up to 2 years | | |  |
| **New Treatment** |  |  |  |
| - epcoritamab + lenalidomide | 18 | 48 weeks |  |

**Part 1 – Main Information Sheet**

| 1. **What is the purpose of the REFRACT study?** |
| --- |

Follicular lymphoma (FL) is a slow growing but incurable cancer of the immune system. Treatment can control the disease, sometimes for several years, but most people need more treatment when the disease returns (relapses) and some people don’t respond to initial treatment as well as hoped (refractory disease).

In these instances, treatment with chemotherapy or lenalidomide in combination with an antibody treatment is the standard of care, but the choice of chemotherapy is not standardised and these treatments may cause unwanted side effects. There are many active and safe non-chemotherapy drugs in development, but no research has been performed yet to compare these drugs to the current standard of care, to tell us if these are better.

This study will investigate three experimental non-chemotherapy treatments (one after the other in three treatment rounds), and compare them to standard of care. This information sheet is for round 1, where we will determine if an experimental non-chemotherapy treatment, epcoritamab combined with lenalidomide, is better than the current standard of care of chemotherapy or lenalidomide combined with an antibody treatment.

Epcoritamab is a drug known as a bispecific antibody, which works by redirecting the immune system to destroy lymphoma cells. Epcoritamab is not currently licenced for use and therefore not available in standard of care. Lenalidomide is an immunomodulatory agent that works by modulating different components of the immune system to kill abnormal cells. Lenalidomide is licenced for use in the UK and available as a treatment option for relapsed or refractory FL (rrFL) in combination with rituximab.

Early studies of epcoritamab suggest that it works at least as well as standard of care treatment, with potential benefits including fewer side effects and more convenient delivery (may be received as an injection compared to some chemotherapy treatments which are via infusion over a few hours). Combining epcoritamab with lenalidomide can enhance the effects of both drugs, leading to more efficient destruction of lymphoma cells.

The main aim of round 1 of the REFRACT study is to compare epcoritamab and lenalidomide treatment to standard of care treatment and gather more information on how effective it is. This will be done by looking at how each patients disease responds to treatment after 24 weeks via a PET-CT scan (see section 5 for more information). Other longer-term aims are to compare how safe the treatments are and how long they are effective, for example how long until patients require another treatment. We will also collect information on quality of life to compare between the treatments.

| 1. **Why have I been invited to take part?** |
| --- |

Your doctor has invited you to consider taking part because you have been diagnosed with rrFL.

Approximately 284 patients across at least 25 hospitals in the UK will participate in all rounds of the study, which is expected to be open to new patients for a total of five years. Round 1 will be open to new patients for two and a half years and 126 patients will participate. The study is expected to last for 10 years, from when it first opens to new patients, until the final patient in round 3 has had treatment and completed follow up. We will collect information (data) on how each patient is doing every year until the end of the study, including quality of life questionnaires every 6 months. Therefore, if you agree to participate, you will be part of the study for up to 10 years. This information will be collected as part of your routine follow-up and no additional visits or tests/procedures are required specifically for the trial.

| 1. **How do I enter the study?** |
| --- |

The first step is to decide whether you want to take part in the study. Your doctor will describe the study and talk through this information sheet with you. This information sheet is yours to keep. If you choose to enter the REFRACT study, you will be invited to sign an Informed Consent Form to show that you understand what is involved.

The original signed Informed Consent Form will be kept in the REFRACT Investigator Site File (the study file kept by your hospital), one copy will be placed in your hospital notes, one copy will be given to you for your records, one copy will be sent to the REFRACT Trial Office and one copy will be sent to the laboratory at the Liverpool Biobank (based at the University of Liverpool) where the samples you provide as part of this study will be stored (please see the section “What samples will be collected?” for more information).

| 1. **Do I have to take part?** |
| --- |

No, participation in this study is entirely voluntary. If you decide not to take part, your treatment and standard of care will not be affected in any way and your doctor will discuss your alternative treatment options with you. If you consent to participate, you are still free to withdraw from the study at any time without giving a reason. Any data or samples collected up until the point of withdrawal will be retained and analysed.

| 1. **What will happen if I decide to take part?** |
| --- |

#### Consent and Screening

If you decide to take part in this study, we will ask you to give your written informed consent and the following tests will be performed to make sure you are suitable for the study. Unless stated otherwise, you would have most of these tests as part of your routine care, whether you take part in the study or not.

- A medical history, including medicines you currently take, and those you have taken in the past
- A physical examination including lymph node examination
- Vital signs assessment to measure your weight, height, temperature, blood pressure, breathing rate, oxygen saturation, pulse, and ECOG performance status (assessment of how your disease affects your daily living abilities) to assess your well-being before entering the study
- A pregnancy test (if you are a female of child-bearing potential)
- Blood tests – these tests are performed to check that you are well enough to begin study treatment and will include a full blood count, assessment of liver and kidney function, tests to look for any viruses in your blood (such as Hepatitis and HIV), coagulation (to see how fast your blood clots) and immunoglobulins (to measure the amount of antibodies in your blood).
- Bone marrow aspirate and biopsy – an aspirate is when some liquid bone marrow is taken via a syringe and a biopsy (called a trephine) is the removal of a small sample of core bone marrow tissue. This will only be performed if your doctor feels this is necessary. This will assess the status of your disease. A sample of the aspirate will be sent to the Liverpool Biobank (more information about this is provided in the “What samples will be collected?” section). If you have a bone marrow procedure, the aspirate and trephine biopsy will be collected at the same time.
- Tumour biopsy – a tumour biopsy is required before you start study treatment to confirm the diagnosis of rrFL. If you have recently had a tumour biopsy, we will collect archived (stored) tissue from your biopsy. If you have not had a tumour biopsy within 3 months of starting the study or your doctor is concerned that your lymphoma is growing quickly, you will have a tumour biopsy during screening.

The procedure usually involves inserting a needle into your lymph node under local anaesthetic and removing a sample of tissue, under guidance of an ultrasound scan. This takes approximately 10-15 minutes. The needle is then removed and a bandage is put onto the area. Your doctor will tell you which lymph node they will take the sample from.

In about 10% of cases, your doctor may need to perform this procedure under the guidance of a CT scan. This will help your doctor identify the most appropriate area of your lymph nodes to take tissue from. Very rarely, the biopsy may need to be done during an endoscopy (camera test) or a small operation.

- PET-CT scan (whole body)
  - **CT scans**

A CT scan (or CAT scan) takes a series of X-rays of the body from different levels. A computer puts the X-rays together to form a picture. Before the scan, you have a drink or an injection into the bloodstream of a dye called contrast medium. The contrast shows up body tissues more clearly on the scan. The scan itself can take 30 minutes. To have the scan you lie on a narrow bed that moves through the scanner. It is important that you lie as still as possible while the scan is being done. It shouldn’t be at all painful. If you begin to feel unwell or want some help, you will have a buzzer that you can press to get attention. The staff doing the scan will be able to see you at all times.

- - **PET-CT scan (sometimes just called PET scan)**

PET Scans or **P**ositron **E**mission **T**omography scans are a type of scan that can show body tissue activity, as well as the size and distribution of abnormal areas. As part of your PET scan you will have an injection of a radioactive version of glucose, also known as 18F-FDG. The amount of radiation is small and only stays in the body for a few hours.

When 18F-FDG is injected into your body it travels to places where glucose is used for energy. It shows up lymphoma because it uses glucose more avidly than normal tissue. After you have the injection you rest for about an hour. This allows the radioactive tracer to spread through the body. The scan itself can take up to an hour and is similar to having a CT scan described above. The whole PET-CT scan should take about two hours.

You may undergo PET-CT scans and potentially CT scans (to assist your clinicians with your biopsies) as part of this study. Whilst such scans are common and taken as "standard of care" if you were not in a study such as this, some of the scans may be additional for the study purposes. PET-CT and CT and scans use ionising radiation to form images of your body. Ionising radiation can cause cell damage that may, after many years or decades, turn cancerous. The chances of this happening to you as a consequence of taking part in this study are extremely low (less than 0.5%).

Your appointment card should give you details about what you need to do to prepare for your scan. After your scans you should feel fine and will be able to go back to your normal activities. Following your scan you may be advised to avoid contact with pregnant women, babies and children for up to 6 hours as a precaution due to the radiation you received as part of the scan.

- Analysis of your urine – a common test done to check overall health. Following this test (and pregnancy test if applicable), your urine will be disposed of locally at your hospital
- Electrocardiogram (ECG) used to check your heart's rhythm and electrical activity through sensors attached to the skin
- Neurological assessment – this will be performed only if you are randomised to receive epcoritamab and lenalidomide (see the “Randomisation” section below for further information). This assessment involves checking your cognitive (brain) function by asking you to perform simple tasks such as naming objects and writing a sentence. This is expected to take 5 minutes to complete
- Quality of life assessment with 2 standardised questionnaires (called EQ-5D-5L and FACT-Lym) (more information about this is provided below under “Quality of Life” on page 8).

The above assessments will also be performed at various time points throughout your treatment. Please refer to the “Summary of trial assessments” on pages 10-13.

**Randomisation**

This study is a randomised, controlled clinical trial. Sometimes we don’t know which way of treating patients is best. To find out, we need to compare different treatments. We put people into groups and give each group a different treatment – these groups are called arms. The results are compared to see if one treatment is more favourable in terms of control of the disease and the side effects experienced. To try to make sure the arms are similar to start with (in other words, the patient groups are matched so that the only difference between arms is the treatment received), each participant is put into an arm at random by a computer. Neither you nor your doctor can choose which arm you will be assigned. This process is called randomisation. In REFRACT round 1, patients will be randomised 50/50 to receive standard of care treatment or epcoritamab and lenalidomide – this is called 1:1 randomisation.

**Treatment**

Epcoritamab and lenalidomide

Treatments will initially be given in 4 week (28 day) cycles. This treatment consists of:

- Epcoritamab given via a single injection into the skin (subcutaneous), on days 1, 8, 15 and 22 of the first and second cycles, followed injections on day 1 only for the remaining 10 cycles, and
- Lenalidomide capsules (20mg) taken every day for 3 weeks, then a week of no capsules, for 12 cycles. The capsules should be taken orally (by mouth) at about the same time on the scheduled days and should not be opened, broken or chewed. The capsules should be swallowed whole, preferably with water, either with or without food. It is recommended to press only on one end of the capsule to remove it from the blister thereby reducing the risk of capsule deformation or breakage.

If less than 12 hours has elapsed since missing a dose, take the dose. If more than 12 hours has elapsed since missing a dose at the normal time, do not take the dose, but take the next dose at the normal time on the following day.

Treatment with epcoritamab and lenalidomide will continue for up to 12 cycles, or until disease progression (worsening of lymphoma on a scan or by symptoms and blood tests), the development of unacceptable side effects or withdrawal from the study for any reason if this occurs sooner.

Because of the potential risk of epcoritamab and lenalidomide causing your immune system to become over activated you will receive some supportive treatment, including steroids, antihistamine and anti-pyretic (such as paracetamol), for a minimum of the first cycle. Steroids will be given for 4 days starting on each day of epcoritamab injection (e.g. days 1-4, 8-11 etc) and an antihistamine and anti-pyretic will be given on each day of epcoritamab injection. Your doctor will discuss with you if you require this supportive treatment beyond the first cycle.

Standard of care treatment

If you are assigned to standard of care chemotherapy and antibody treatment, you are likely to receive one of these different standard options:

- R-CHOP (rituximab, cyclophosphamide, doxorubicin, vincristine and prednisolone) – given over 4 months (6 21-day cycles). Rituximab, cyclophosphamide, doxorubicin and vincristine are given via an infusion on day 1 of each cycle and prednisolone will be given as tablets to take on days 1-5 of each cycle. Rituximab maintenance may be administered every 2 months for 2 years.
- R-CVP (rituximab, cyclophosphamide, vincristine and prednisolone) – given over 4 months (6 21-day cycles). Rituximab, cyclophosphamide and vincristine are given via an infusion on day 1 of each cycle and prednisolone will be given as tablets to take on days 1-5 of each cycle. Rituximab maintenance may be administered every 2 months for 2 years.
- Bendamustine and rituximab – given over 6 months (6 28-day cycles). Rituximab will be given via an infusion weekly for the first cycle then on day 1 of each remaining cycle. Bendamustine will be given via an infusion on days 1 and 2 of each cycle. Rituximab maintenance may be administered every 2 months for 2 years.
- Bendamustine and obinutuzumab – given over 6 months (6 28-day cycles) followed by a maintenance period of obinutuzumab every 2 months for 2 years. Both drugs will be given via infusion; bendamustine on days 1 and 2 of each cycle for 6 cycles and obinutuzumab on day 1, 8 and 15 of cycle 1, day 2 of cycles 2-6, then once every 2 months for 2 years.
- Lenalidomide and rituximab – given over 1 year (12 28-day cycles). Rituximab will be given via an infusion weekly for the first cycle then on day 1 of each cycle for 5 cycles. Lenalidomide capsules will be taken on days 1-21 of each cycle for 12 cycles.

Your doctor will discuss these options with you, and decide which is best for your disease. Your doctor will provide you with written information about the standard of care treatment combination that has been selected for you.

To help control the side effects of treatment you will also receive some additional medication as follows:

- If you receive rituximab you will also receive a steroid, antihistamine and antipyretic (a drug to reduce fever for example paracetamol) on each day you have rituximab.
- If you receive obinutuzumab you will also receive a steroid, antihistamine and antipyretic (a drug to reduce fever for example paracetamol) on the day you have your first dose of obinutuzumab. For further doses of obinutuzumab you will receive a minimum of an antipyretic before each dose. You may also need to receive a steroid and/or antihistamine. If this is required your doctor will discuss this with you.

**Quality of Life**

As part of the REFRACT trial, we are very interested in how your treatments might affect your Quality of Life (QoL). We think it is very important to find out how patients taking part in the REFRACT trial feel, both emotionally and physically, and to study any side effects in detail.

You will, therefore, be asked to fill out 2 questionnaires at screening, at the start of cycle 3 of treatment, at the end of your treatment and then every 24 weeks (6 months) until the end of the study (up to 10 years). The questionnaires will take approximate 15 minutes to complete.

The following tables show a summary of all the assessments you can expect to have during this trial. There are 2 tables, one for patients randomised to epcoritamab and lenalidomide and one for patients randomised to standard of care, as the assessments and visit time points can vary between the treatments.

**Summary of trial assessments- epcoritamab and lenalidomide patients**

| **Assessment** | **Screening**  (within 28 days of trial entry) | **Treatment Phase** | | | | | | | | | | | **Follow up** | |
| --- | --- | --- | --- | --- | --- | --- | --- | --- | --- | --- | --- | --- | --- | --- |
|  |  | **Cycle 1** | | | | **Cycle 2** | | | | **Cycle 3-12** | **24 weeks from treatment start** | **End of treatment** | **60 days post treatment** | **24 weekly** |
|  |  | D1 | D8 | D15 | D22 | D1 | D8 | D15 | D22 | D1 |  |  |  |  |
| Informed consent | x |  |  |  |  |  |  |  |  |  |  |  |  |  |
| Medical history | x |  |  |  |  |  |  |  |  |  |  |  |  |  |
| Vital signs, height and weight^1^ | x | x | x | x | x | x | x | x | x | x |  | x |  |  |
| Physical examination | x | x | x | x | x | x | x | x | x | x |  | x |  |  |
| Neurology assessment |  | x |  | x |  | x |  | x |  | x |  | x |  |  |
| ECG | x | If your doctor deems this to be necessary | | | | | | | | | | |  |  |
| ECOG Performance Status | x |  |  |  |  | x |  |  |  | x |  | x |  |  |
| Pregnancy test^2^ | x | x |  |  |  | x |  |  |  | x |  | x |  |  |
| **Blood tests** |  | | | |  | | | | | | | | | |
| Full blood count and organ function tests | x | x | x | x | x | x | x | x | x | x | x | x | x |  |
| Coagulation | x | If your doctor deems this to be necessary | | | | | | | | | | |  |  |
| Virus screen | x |  |  |  |  |  |  |  |  |  |  |  |  |  |
| Immunoglobulins | x |  |  |  |  |  |  |  |  |  |  | x |  |  |
| Urinalysis | x | If your doctor deems this to be necessary | | | | | | | | | | |  |  |
| PET-CT whole body^4^ | X^3^ |  |  |  |  |  |  |  |  |  | x |  |  |  |
| **Samples^5^** |  | | | |  | | | | | | | | | |
| Tumour biopsy^4^ | X^6^ | If your doctor deems this to be necessary | | | | | | | | | | | | |
| Bone marrow biopsy/aspirate (if necessary)^4^ | x | If your doctor deems this to be necessary | | | | | | | | | | | | |
| Research blood samples^4^ |  | x |  |  |  |  |  |  |  | x (cycle 4 day 1) | x | x |  | x^7^ |
| Saliva |  | x |  |  |  |  |  |  |  |  |  |  |  |  |
| Quality of Life Questionnaires (EQ-5D-5L and FACT-Lym) | x |  |  |  |  |  |  |  |  | x (cycle 3) | x |  |  | x |

1. Height will be taken at screening only
2. Pregnancy test in women of childbearing potential only. Screening pregnancy test must be taken within 7 days of treatment start
3. May be performed within 6 weeks of treatment start
4. Also to be performed if your disease progresses
5. See section below “What samples will be taken?” for further information
6. To be taken within 3 months of treatment start
7. Samples to be taken 12 months after starting treatment

**Summary of trial assessments- standard of care treatment**

| **Assessment** | **Screening** (within 28 days of trial entry) | **Treatment Phase** | | | | | | | | | | **Follow up** | |
| --- | --- | --- | --- | --- | --- | --- | --- | --- | --- | --- | --- | --- | --- |
|  |  | **Cycle 1 Day 1** | **Cycle 2 Day 1** | | **Cycle 3 Day 1** | **Cycle 4 Day 1** | **Cycle 5 Day 1** | **Cycle 6 Day 1** | **End of treatment** | **24 weeks from treatment start** | | **60 days post treatment** | **24 weekly** |
| Informed consent | x |  |  | |  |  |  |  |  |  | |  |  |
| Medical history | x |  |  | |  |  |  |  |  |  | |  |  |
| Vital signs, height and weight^1^ | x | x | x | | x | x | x | x | x |  | |  |  |
| Physical examination | x | x | x | | x | x | x | x | x |  | |  |  |
| ECG | x |  | | | If your doctor deems this to be necessary | | | | |  | |  |  |
| ECOG Performance Status | x | x | x | | x | x | x | x | x |  | |  |  |
| Pregnancy test^2^ | x | x | x | | x | x | x | x | x |  | |  |  |
| **Blood tests** |  | | |  | | | | | | | | | |
| Full blood count and organ function tests | x | x | x | | x | x | x | x | x | x | | x |  |
| Coagulation | x | If your doctor deems this to be necessary | | | | | | | |  | |  |  |
| Virus screen | x |  |  | |  |  |  |  |  |  | |  |  |
| Immunoglobulins | x |  |  | |  |  |  |  | x |  | |  |  |
| Urinalysis | x | If your doctor deems this to be necessary | | | | | | | |  | |  |  |
| PET-CT whole body^3^ | X^4^ |  |  | |  |  |  |  |  | x | |  |  |
| **Samples^5^** |  | | |  | | | | | | | | | |
| Tumour biopsy^4^ | x^6^ | If your doctor deems this to be necessary | | | | | | | | | | | |
| Bone marrow biopsy/aspirate (if necessary)^4^ | x | If your doctor deems this to be necessary | | | | | | | | | | | |
| Research blood samples^4^ |  | x |  | |  | x |  |  | x^7^ | x^7^ | |  | x^8^ |
| Saliva | x |  |  | |  |  |  |  |  |  | |  |  |
| Quality of Life Questionnaires (EQ-5D-5L and FACT-Lym) | x |  |  | | x |  |  |  | x |  |  |  | x |

1. Height will be taken at screening only
2. Pregnancy test in women of childbearing potential only. Screening pregnancy test must be taken within 7 days of treatment start, subsequent tests are only required for female patients of childbearing potential taking lenalidomide
3. May be performed within 6 weeks of treatment start
4. Also to be taken if your disease progresses
5. See section below “What samples will be taken?” for further information
6. To be taken within 3 months of treatment start
7. If these time points coincide, only one sample will be taken
8. Samples to be taken 12 months after starting treatment

| 1. **What samples will be taken?** |
| --- |

The following samples will be taken for research purposes. All samples are mandatory as part of the study unless stated otherwise. Samples will be analysed as part of different work projects which aim to;

1. Look at tumour cells in different patients to see if there is any difference in the cells of patients who respond well to treatment and those who don’t. It is hoped that this will enable researchers to predict which patients are going to respond well to treatment, so treatment can be more targeted to individual patients in the future.
2. Look for any specific features of the samples (for example the make-up of the immune cells) that might predict if a patient is going to relapse or not respond to treatment and how these features change during the course of treatment. It is hoped that in the future this will enable researchers to identify which patients are more likely to relapse before this happens so that doctors can take action sooner.

**Blood samples**

We will obtain some additional blood samples before you start treatment, at the beginning of cycle 4, at 24 weeks (6 months) after treatment start, at the end of your treatment (if this coincides with the 24 week time point, only 1 sample will be requested), and if your disease progresses (worsens). Where possible, these samples will be taken at the same time as the routine samples so that you will not have to undergo additional procedures. We will collect 40mls (8 teaspoons) of blood at each time point.

**Saliva**

We will take a saliva sample before you start treatment. This is an additional sample to what you would provide in routine care. The test is simple and non-invasive, you will be asked to spit into a tube 15-20 times.

**Bone marrow aspirate**

In addition to the bone marrow aspirate sample collected at screening, we will also collect additional aspirates at any other point during the trial if you undergo a bone marrow aspiration recommended by your doctor, or if your disease progresses. These samples will not require you to have any additional procedures. We will collect 6mls (just over 1 teaspoon) of bone marrow aspirate at each time point.

**Tumour biopsy**

In addition to the tumour biopsy collected at screening we will also collect leftover tissue if available at any other point during the trial if you undergo a biopsy or surgery recommended by your doctor, or if your disease progresses. These samples will not require you to have any additional procedures. Additionally, we would also like to collect any left over tissue from a tumour biopsy you may have had at your initial diagnosis (when you were first diagnosed with FL), this is optional.

Samples collected will be stored at the Liverpool Biobank then transferred to a laboratory for analysis during the course of the trial. The sample analysis will take place at 3 laboratories based at the University of Cambridge, Barts Cancer Institute and Guy’s Cancer Centre (London).

| 1. **What will happen to the samples at the end of the study?** |
| --- |

We will need to keep the samples collected until the end of the study to carry out the research.

If you agree, we would like to keep any leftover samples after the study has finished to allow further research, for example to test new treatments or to design new tests that predict which patients are most likely to respond well to this treatment. Donation of your samples for existing (outside of the REFRACT study) or future research is optional, and you can still enter the study without agreeing to this.

The Liverpool Biobank is a tissue bank, fully accredited by the Human Tissue Authority, and already collects and stores similar samples from many patients with blood cancers taking part in trials. Any samples that are stored may be analysed in an existing study or in the future as part of a new research study. This may involve sending the samples abroad. Any research carried out on the samples will need to have ethical approval.

If you do not agree to donate your samples for other existing or future research, these will be safely destroyed at the end of the study.

| 1. **PET-CT scan study** |
| --- |

As well as reporting the outcome of treatment, we will collect the information from your PET-CT scans for research. This research is aimed at finding out if scans can provide more information about your response to treatment, and whether the information can be used to predict the long-term outcome of your lymphoma. The research will be carried out at King’s College London and Guy’s and St Thomas’ PET Centre and may not be done in real-time, i.e. the results of the research will not be fed back to your doctor in order to inform any of your current or future treatment. Your scans will however be reviewed locally by your hospital and this review may be used to inform your treatment management.

| 1. **What are the possible benefits of taking part?** |
| --- |

By taking part in this study, you will receive active treatment for your lymphoma. However, there is no guaranteed additional benefit of taking part in this study because we do not yet know which of the two treatment approaches is better. Epcoritamab has shown promise in early trials and so may be an important treatment for rrFL, alone or in combination with lenalidomide as being tested in this trial; it may, however, not provide any overall benefit compared with current standard of care treatment. The information gained from this study will help inform treatment for other people in the future.

| 1. **What are the possible disadvantages and risks of taking part?** |
| --- |

**Bone marrow aspirate and biopsy**

This procedure will only be done if deemed necessary by your doctor. The procedure may be painful, but you will be given a local anaesthetic to numb the area before any sample is taken. However, it may be a little uncomfortable afterwards. Please tell your doctor if you have any pain and you will be given painkillers. The area will be covered with either a sticky plaster or a gauze pad. There may be a small amount of bleeding which is perfectly normal, but the doctor or nurse will make sure this has stopped before you go home. If the site does start to bleed again, press on the area with a clean cloth or handkerchief. By pressing on the area, this will help your blood to clot and the bleeding to stop. Sometimes patients require additional measures to tolerate the biopsy, such as mild sedation. Discuss this with your medical team if you feel you would require this.

**CT, PET and PET-CT- radiation risks**

As part of this study, you will have whole body PET-CT scans. These tests are to look at the extent of disease and how you are responding to treatment. Taking part in this trial may mean one additional PET-CT scan prior to treatment (note some patients may have received this as part of their routine care). These tests use ionising radiation, which is thought to be associated with a very small increased risk of developing a second cancer in the future. At present, there are no better ways of imaging the body that avoid radiation, and the tests are performed to evaluate the extent of your lymphoma before and after treatment, to see how well treatment has worked.

**Side effects of treatment**

We want to try and improve the outcome for patients with rrFL and believe that epcoritamab and lenalidomide may improve outcomes in patients. However, there is the possibility that this may not show any benefit over the current UK standard of care treatment, or that the side effects might be worse. The known common side effects of epcoritamab, lenalidomide and standard of care treatments are outlined below. You will be monitored throughout treatment for any side effects and given medication as required to help with any side affects you experience. It is important that you tell your nurse or doctor of any new symptoms you are experiencing.

Eporitamab

Epcoritamab is an antibody designed to simultaneously recognise lymphoma cells and the cells of your immune system (called T-cells and B-cells), bringing them together to destroy lymphoma cells. Epcoritamab works together with lenalidomide to kill lymphoma cells. However, they can also cause side effects if the immune system targets normal cells. Side effects occur when healthy cells become damaged. Side effects of epcoritamab include:

The most common (occurring in just over half of patients) side effect of epcoritamab is cytokine release syndrome (CRS) and associated fever. CRS occurs when the immune system responds to the drugs more aggressively than normal and as well as fever, other symptoms include chills, tiredness, nausea, vomiting, diarrhoea and headaches. The majority of CRS cases have been mild with few patients experiencing more severe CRS (requiring intervention and hospitalisation). Most instances of CRS occur during the first cycle of treatment. You will be given medication to help prevent CRS for at least the first cycle of treatment. Other common (occurring in more than 1 in 10 patients) side effects that are known to occur include:

- Tachycardia (a fast heart rate)
- Hypotension (low blood pressure)
- Headache and dizziness
- Chills
- Fatigue
- Hypoxia (low oxygen levels in the blood) and shortness of breath
- A reaction where epcoritamab is injected into the skin including swelling, pain, rash, bleeding or redness
- Gastrointestinal side effects such as nausea, vomiting, abdominal pain, diarrhoea and constipation
- Low blood count levels such as low red blood cells, white blood cells and platelets
- Confusional state
- Low phosphate and potassium
- Raised blood sugar
- Infections including urinary tract infection and respiratory tract infection
- Joint stiffness and back pain
- Cough
- Pneumonia
- Leg swelling (fluid retention)
- Liver function test increases
- Rash
- Excess sweating
- Chest pain
- Inflammation of the pancreas
- Adrenal insufficiency (when the adrenal glands don’t make enough of the hormone cortisol)
- Pleural effusion (excess fluid on the lungs)
- Depressed level of consciousness

A less common side effect (occurring in less than 1 in 10 patients) of epcoritamab is immune effector cell-associated neurotoxicity (ICANS) - this is a neurological disorder related to CRS. Symptoms can include tremors, lethargy and difficulty speaking, writing and spelling. Symptoms are expected to last less than 1 week.

The above side effects are most likely to occur in the first 2 cycles of treatment and are likely to last less than 3 days.

Lenalidomide (applicable for patients on epcoritamab + lenalidomide, or standard of care patients on lenalidomide + rituximab)

The table below shows side effects with lenalidomide.

All side effects listed are common (occurring in more than 1 in 100 but less than 1 in 10 patients), unless ‘very common’ (occurring in more than 1 in 10 patients) is stated, and include (but are not limited to):

| **Category** | **Severity** | |
| --- | --- | --- |
|  | Mild/moderate^1^ | Severe/life-threatening^2^ |
| Infections | Upper respiratory tract infection (very common), sinusitis | Pneumonia, sepsis, bronchitis, urinary tract infection, lung infection, gastroenteritis, cellulitis (skin infection) |
| Secondary cancers or cysts/polyps | Tumour flare (very common) – a temporary reaction to treatment where the tumour can increase in size, symptoms include painful and swollen lymph nodes or spleen, slight fever, bone pain, and skin rash | Secondary cancers of the skin |
| Blood disorders (very common) | Decreased blood count levels including low white and/or red blood cells and low platelets | |
| Nutrition disorders | Decreased appetite (very common), dehydration | Decreased potassium or phosphate levels, or increased calcium or uric acid levels |
| Psychiatric disorders | Depression, insomnia |  |
| Neurological symptoms | Headache and dizziness (very common), fainting, distorted sense of taste, peripheral sensory neuropathy (damage to nerves causing weakness, numbness and pain, usually in the hands or feet) | Fainting |
| Vascular (circulatory system) disorders |  | Low blood pressure, pulmonary embolism- a blood clot that forms in a blood vessel in the body then travels to a lung |
| Respiratory disorders | Cough (very common), voice problems such as hoarseness and throat pain | Shortness of breath |
| Gastrointestinal disorders | Nausea (very common), vomiting (very common), dry mouth, indigestion (very common) | Abdominal pain, diarrhoea, constipation, inflamed/sore mouth |
| Skin disorders | Dry skin, night sweats | Rash, itchy skin |
| Muscle/bone problems | Spasms (very common) , back pain (very common, joint stiffness (very common, aches and pains | Neck pain, muscular weakness |
| Renal disorders |  | Acute kidney injury – this is where the kidneys do not function properly, symptoms can include swelling in the legs or ankles, tiredness or shortness of breath |
| General disorders | Fever (very common, peripheral oedema (swelling due to excess fluid) (very common, weight loss, abnormal liver function tests (very common | Tiredness/weakness |

1 Mild- no symptoms or mild symptoms, intervention not required. Moderate- minimal intervention required

2 Severe- medically significant but not life-threatening, hospitalisation may be required. Life-threatening- urgent intervention required. If any of these side effects are experienced, please see the ‘Emergencies’ section at the end of this information sheet

|  |
| --- |

Standard of Care Treatments

Your doctor will provide you with information on side effects you may expect with the specific treatment they decide to give you. Here we have included common side effects from the classes of drugs that may be included in your treatment if randomised to standard of care.

*Chemotherapy*

The chemotherapy drugs included in this study are bendamustine, cyclophosphamide, vincristine and doxorubicin.

Chemotherapy destroys tumour cells, but can also damage normal healthy cells in the body. Side effects occur when healthy cells become damaged.

Common side effects of chemotherapy drugs include:

- Hair loss, but it will grow back after the chemotherapy has finished
- Nausea (feeling sick) and vomiting
- Reduced bone marrow function

The bone marrow makes red blood cells, white blood cells and platelets.

- - Red blood cells carry oxygen. If these are low, you may be tired or pale and need a blood transfusion.
  - White blood cells fight infection. If these are low, you will be at risk of developing an infection. If you develop a fever, admission to hospital will be necessary for intravenous antibiotics (through a drip).
  - Platelets help stop bleeding and bruising. If these are low, a platelet transfusion can be given.

The effect on the bone marrow is temporary. Before each cycle of chemotherapy, blood tests will be done to check that your bone marrow function has recovered enough to continue.

Other side effects include a sore mouth, dry skin, constipation and diarrhoea. These are temporary. Your doctor or a dietician will be able to advise you if necessary. Medication is available to help manage some of the side effects.

There are other side effects of chemotherapy, some of which can be serious or life-threatening, and your doctor will discuss these with you.

Chemotherapy can also have long-term effects such as a reduction in fertility and an increased risk of developing a second cancer after the completion of treatment. Your doctor will discuss these side effects with you and the possibility of sperm or egg preservation.

*Antibody treatments targeting CD20*

Antibody treatments target proteins on the surface of the lymphoma cells, identifying them as cells for your immune system to kill. Common side effects of rituximab and obinutuzumab - the two CD20-directed antibodies used in combination with chemotherapy to treat rrFL - include:

- Sore/tender joints (common)
- Weakness (very common)
- Fever, vomiting, or a rash and/or shaking during infusion (very common)
- Headaches (very common)
- A reduction in the number of antibody-producing cells in the body (very common)
- Infections (very common)
- Low blood counts (very common)

Common side effects occur in more than 1 in 100 but less than 1 in 10 patients and very common side effects occur in more than 1 in 10 patients.

These are temporary. Your doctor or a dietician will be able to advise you if necessary. Medication is available to help manage some of the side effects.

*Prednisolone*

Some standard treatments include prednisolone which is a steroid.

Common side effects (affecting more than 1 in 100 but less than 1 in 10 people) include:

- Infections
- Hormone disorders which may include symptoms such as weight gain or loss, muscle weakness, lack of appetite, low blood pressure
- Low potassium levels
- Weakening of bones (osteoporosis)
- Swelling caused by excess fluid in the body (oedema)
- High blood pressure
- Thinning of the skin/ impaired wound healing

Less common side effects (affecting more than 1 in 1000 but less than 1 in 100 people) include:

- Eye disorders such as cataract or glaucoma (damage to the optic nerve)
- Return of previous mental disorders (for example depression)

Risks of severe COVID-19 are likely to be increased during treatment and the response to COVID-19 vaccines may be reduced. This will be the same whether you receive standard treatment or epcoritamab and lenalidomide.

**Harm to the unborn child**

Information for Women

All of the drugs in this study may cause harm to an unborn child if administered during pregnancy. There is little or no information on the effects on the child when breast feeding during treatment with any of the drugs in this study (except for prednisolone which is unlikely to cause harm during breastfeeding).

You cannot take part in this study if you are pregnant, breast-feeding, planning to become pregnant or to make an egg donation. If you are a female who can become pregnant, you will be asked to take a pregnancy test prior to starting study treatment and you may be asked to take further tests throughout treatment depending on the drugs you are taking (please see the summary of trial assessments tables above).

If you decide to take part in this study, you must agree to use at least one highly effective method of contraception plus a barrier method of contraception (see box below) during the trial treatment and for a period of time after the last dose of treatment (please see the table below).

If you become pregnant while receiving study medication, or within the time periods specified below depending on which drugs you were taking, you must tell your doctor right away and any study medication you are taking will be discontinued. Your doctor will explain how to safeguard your health and the health of your baby. If you agree, we will collect information related to the progress of your pregnancy and its outcome that is relevant to the study. This may include information related to your health, the date of conception, the course and outcome of your pregnancy and any medical treatments that you receive.

Information for Men

If you were to father a child, the treatments you will receive as part of this study may be harmful to the unborn child.

If your partner might become pregnant, you/your partner must agree to use at least one highly effective method of contraception and one barrier method of contraception (see box below) during the trial treatment and for a specified amount of time following treatment depending what drug you were taking (please see the table below). Your doctor will talk to you about potential sperm donation before you start treatment and you should not be involved in sperm donation during treatment. If your partner becomes pregnant during the course of the study, or within the time periods specified below depending on what drug you were taking, we would ask you to tell your study doctor immediately and your doctor will ask you and your partner for permission to collect information about the pregnancy and the child. Your doctor will explain to you and to your partner how to safeguard your partner’s health and the health of the baby.

The table below shows how long you should continue to take contraception depending on which drug/s you have received. The guidance for each drug may be different for men and women.

| **Drug** | **Duration from last dose** | **Male/female** |
| --- | --- | --- |
| Epcoritamab | 12 months | Males and females |
| Rituximab | 12 months | Females |
| Bendamustine | 6 months | Males |
|  | 3 months | Females |
| Cyclophosphamide | 6 months | Males |
|  | 12 months | Females |
| Doxorubicin | 6 months | Males and females |
| Lenalidomide | 7 days | Males |
|  | 4 weeks | Females |
| Obinutuzumab | 18 months | Females |
| Vincristine | 4 months | Males |
|  | 7 months | Females |

| Highly effective methods of contraception include:  combined pill, progestogen-only hormonal contraception associated with inhibition of ovulation, intrauterine device (IUD), intrauterine hormone-releasing system (IUS), bilateral tubal occlusion (surgery to cut/block fallopian tubes), vasectomised partner or abstinence.  Barrier methods of contraception include:  Male/female condom, diaphragm, cervical cap, contraceptive sponge or spermicide. |
| --- |

**What additional medications should I avoid?**

**It is very important that you tell the study doctor about all medications, supplements, or herbal medicine that you are taking now and during the study.** Even herbal medicines and other alternative treatments can interact with the medicines you will be given, and these interactions could be dangerous. St. Johns Wort should be avoided in patients receiving R-CHOP or R-CVP. Your doctor will discuss with you if there are any other medications that you should avoid.

You should also not receive any other chemotherapy, immunotherapy or other experimental therapy whilst on the trial.

# **Part 2 – Additional Information**

| 1. **Will I be paid to take part?** |
| --- |

You will not receive any money for taking part in this study and unfortunately travel expenses cannot be reimbursed by the study organisers. However, some hospitals may offer travel expenses if you take part in the study and other transport services **may** be available. Please discuss access to these with your medical team.

| 1. **What if there is a problem?** |
| --- |

The main concern of everyone involved with this study is that your treatment is as safe, effective and tolerable as possible. If you have a concern about any aspect of this study, you should ask to speak to your doctor or study nurse who will do their best to answer your questions.

**Complaints**

If you remain unhappy and wish to complain formally, you can do this through the NHS Complaints Procedure. Details can be obtained from your hospital. Additionally, the contact information for your local Patient Advice and Liaison Service (PALS) or equivalent is at the end of this information sheet.

**If you are harmed**

If you are harmed by taking part in this study due to someone’s negligence, then you may have grounds for a legal action for compensation but you may have to pay your legal costs. NHS Trusts have a duty of care to participants whether or not the participant is taking part in a clinical trial and normal National Health Service complaints mechanisms will still be available to you. The University of Birmingham has in place Clinical Trials indemnity coverage for this trial which provides cover for the University’s legal liability for harm caused to participants which comes about through the University’s, or its staff’s, negligence in relation to the design or management of the trial and may alternatively, and at the University’s discretion, provide cover for non-negligent harm to participants. With respect to the conduct of the trial at Site and other clinical care of the patient, responsibility remains with the NHS organisation responsible for the clinical site and is therefore indemnified through NHS Resolution. If you have private medical insurance, you should tell your insurer that you are taking part in research.  They will let you know if it affects your policy. The University of Birmingham is independent of any pharmaceutical company, and as such it is not covered by the Association of the British Pharmaceutical Industry (ABPI) guidelines for participant compensation.

| 1. **What if new information becomes available?** |
| --- |

If we get new information about the treatment being studied your doctor will tell you and discuss whether you should continue in the study. If you decide not to carry on, your doctor will plan for your care to continue. If you decide to continue in the study your doctor may ask you to sign an updated Informed Consent Form. If new information becomes available your doctor might consider that you should withdraw from the study. Your doctor will explain the reasons and arrange for your care to continue. If the study is stopped for any other reason, we will tell you and arrange your continuing care so you receive the best care available.

| 1. **Will my taking part in this study be kept confidential?** |
| --- |

All information collected about you for this study will be subject to the General Data Protection Regulation and Data Protection Act 2018 and will be kept strictly confidential.

All information collected by the Sponsor will be securely stored, on paper and electronically, at the Trial Office at the Cancer Research UK Clinical Trials Unit, University of Birmingham and will only be accessible by authorised personnel associated with the trial. The only people in the University of Birmingham who will have access to information that identifies you will be people who manage the study or audit the data collection process. When you are entered into the study, we will collect your date of birth. You will be given a unique study number and in routine communication between your hospital and the Trial Laboratories (based at the Liverpool Biobank, University of Cambridge, Barts Cancer Institute and Guy’s Cancer Centre (London))., PET centre and Trial Office, you will only be identified by this study number. A copy of your signed consent form will be posted to the Trial Office to ensure that the correct consenting procedure has been carried out. A copy of your signed consent form will also be sent to the Biobank at the University of Liverpool so that they have confirmation that you have consented to your samples being analysed. This will have your name and signature on it. We will also collect your ethnicity. All information collected during the course of the study will be held for a minimum of 25 years from the end of the study.

The NHS will use your name and contact details to contact you about the research study, and make sure that relevant information about the study is recorded for your care, and to oversee the quality of the trial.

Samples taken for research purposes and sent to the Liverpool Biobank, and your PET-CT scans sent to the PET centre will be pseudonymised, meaning they will be identified by your unique study number and date of birth. This information is the minimum needed to make sure that your samples and scans can be identified as yours.

In addition, anonymised data (i.e., with patients’ date of birth removed) from the trial may be provided to other 3rd parties (e.g., pharmaceutical companies or other academic institutions) for research, safety monitoring or licensing purposes. The data may still include your unique study number. This includes sending data to the manufacturers of the drug (Genmab and AbbVie) for safety monitoring purposes. They have the same duty of confidentiality to you as other personnel. These organisations could be within Europe, or outside Europe where the data protection laws may be different. Data sent abroad will not allow you as an individual to be identified.

By taking part in the study, you will be agreeing to allow research staff from the Trial Office at the University of Birmingham to look at the study records, including your medical records that are relevant to this study. It may be necessary to allow authorised personnel from government regulatory agencies (e.g., Medicines and Healthcare products Regulatory Agency (MHRA)), the Sponsor and/or NHS bodies to have access to your medical and research records. This is to ensure that the study is being conducted to the highest possible standards.

From time to time we may be asked to share the trial information (data) we have collected with researchers running other studies in this organisation and in other organisations so that they can perform analysis on the data to answer other important questions about rrFL. These organisations may be universities, NHS organisations or companies involved in health research and may be in this country or abroad. Any such request is carefully considered by the study researchers and will only be granted if the necessary procedures and approvals are in place. This information will not identify you and will not be combined with other information in a way that could identify you. The information will only be used for the purpose of health research, and cannot be used to contact you or to affect your care. It will not be used to make decisions about future services available to you, such as insurance. Under no circumstances will you be identified in any way in any report, presentation or publication arising from this or any other study.

You can withdraw your consent to our processing of your data at any time. Under the provisions of the General Data Protection Regulation (GDPR) 2018, you have the right to know what information the Trial Office has recorded about you. If you wish to view this information, or find more about how we use this information, please contact Legal Services at the address below or email [dataprotection@contacts.bham.ac.uk](mailto:dataprotection@contacts.bham.ac.uk).

Legal Services
University of Birmingham
Edgbaston
Birmingham, B15 2TT

Due to the purposes of the research your data subject rights may be limited, for example you will not be able to change or remove any information we hold about you. Any information request will be reviewed by the Data Protection Officer and Legal Services.

**Involvement of the General Practitioner (GP) /Family Practitioner**

It is important that your GP is kept up to date with any treatment you are receiving. Your GP will be informed that you are taking part in this research study and they will be sent a copy of this information sheet.

| 1. **Will any genetic tests be done?** |
| --- |

Yes. In cancer, the tumour cells have developed a different genome to the healthy cells. There are differences between the DNA from the cancer cells and the healthy cells of the same person. Changes in the DNA from the cancer cells that are not found in the healthy cells are called “somatic mutations”. Comparing the normal and cancer genomes may give clues about ways to treat the cancer

Cancer researchers study DNA to ask questions like:

- Are there any changes in DNA that make an individual more likely to develop a particular cancer? These changes are called “germline variants or mutations”
- Are there any changes in the DNA of the cancer cells that explain where the cancer comes from?
- Do changes in the DNA of the cancer cells tell doctors and patients how a cancer will grow and behave?
- Can changes in the DNA be used to predict which treatment type would be best for which cancer and which patient?
- What genes are active in your cells and how are they affected by the drug you are being treated with?

Researchers will examine DNA from your tumour cells (obtained from the samples you provided in the study). Researchers can use either “targeted sequencing”, in which only a small number of genes are examined or “whole genome sequencing” where the full DNA code is examined.

These tests only look for genetic changes which occurred during your life-time. They do not look for inherited genetic problems and so these results will not have consequences for your family members. Results and data from analysing the DNA taken as part of the result may not be returned to you and may not help with your medical care now or in future.

| 1. **What will happen to the results of the study?** |
| --- |

When the study is complete the results will be published in a medical journal but no individual patients will be identified. If you would like to have a copy of the published results, please ask your study doctor or nurse. Additionally a plain English summary will be published on the Research Summaries section of the Health Research Authority (HRA) website at the end of the study <https://www.hra.nhs.uk/planning-and-improving-research/application-summaries/research-summaries/>

| 1. **Who is organising and funding the research?** |
| --- |

This is an investigator-initiated and investigator-led trial developed and led by the UK National Cancer Research Institute and funded by Cancer Research UK. The drug manufacturer Genmab are providing free epcoritamab for study patients and funding for some of the research on the samples. The trial has been independently peer-reviewed and has been adopted by the NIHR Clinical Research Network Portfolio.

| 1. **Who has reviewed the study?** |
| --- |

This research study has been reviewed by the Cancer Research UK Clinical Trials Unit at the University of Birmingham and by an independent Research Ethics Committee. Research Ethics Committees review all research to protect the safety, rights, wellbeing, and dignity of participants. It has been reviewed and received a Clinical Trial Authorisation by the UK Competent Authority (Medicines and Healthcare products Regulatory Agency - MHRA). It has also been reviewed by the national Health Research Authority (HRA). Clinical and statistical leads at Genmab and Abbvie have reviewed the study, as has the funder, Cancer Research UK.

| 1. **How have patients been involved in this study?** |
| --- |

The Trial Management Group for this study has a patient representative. They have provided advice on the trial design and reviewed this information sheet and helped us to develop it so that it is clear and correct. They will also be involved in the ongoing management of this study.

| 1. **Further information and contact details** |
| --- |

If you have any questions or concerns about your disease or this research study, please discuss them with your doctor. You may also find it helpful to contact the following organisations:

You may also find it helpful to contact the following organisations:

**England**

Your local Patient Advice and Liaison Service (PALs) or equivalent who provide advice and support to patients, their families and their caregivers, website: <http://www.nhs.uk/chq/Pages/1082.aspx?CategoryID=68&SubCategoryID=153>

Or local PALS details, where available:

**Northern Ireland**

In Northern Ireland the Patient Client Council (PCC) can help and support at any stage of the health and social care services complaints procedure. The PCC is an independent body who represent the views of the public in all areas of health and social care. They can also assist you to make a complaint. This is a confidential and free service.

<http://www.patientclientcouncil.hscni.net/>

Telephone: 0800 917 0222

Email: [info.pcc@hscni.net](mailto:info.pcc@hscni.net)

**Scotland**

The Patient Advice and Support Service is an independent service which provides free, accessible and confidential information, advice and support to patients, their caregivers, and families about NHS healthcare in Scotland.

<http://www.patientadvicescotland.org.uk/>

**Wales**

Community Health Councils (CHCs) are independent bodies, set up by law, who listen to what individuals and the community have to say about the health services with regard to quality, quantity, access to and appropriateness of the services provided for them. CHCs can also help, advise and support people who wish to make complaints about NHS services and similar matters. This advice is completely free, independent and confidential.

<http://www.wales.nhs.uk/sitesplus/899/home>

**Sources of information**

**Several patient support groups provide general information and support Follicular Lymphoma patients and their families:**

**Follicular Lymphoma Foundation-** <https://www.theflf.org/>

**Lymphoma Action-** <https://lymphoma-action.org.uk/support-you>

**Blood Cancer UK -** <https://bloodcancer.org.uk/support-for-you/clinical-trials/>

**CancerHelp:** an information service about cancer from Cancer Research UK, Freephone 0808 800 40 40, [www.cancerhelp.org.uk](http://www.cancerhelp.org.uk)

**Macmillan Cancer Support:** Freephone 0808 800 0000, [www.macmillan.org.uk](http://www.macmillan.org.uk)

**CRUK Clinical Trial Database:** <https://www.cancerresearchuk.org/about-cancer/find-a-clinical-trial>

**Emergencies**

**If a medical emergency, related to your treatment for this study occurs while you are at home, you should initially try to contact the haematology unit where you received your treatment (see contact details above). If this is not possible you should go to the accident and emergency (A&E) department at your local hospital. If you are unable to get to the hospital you should contact your GP who will already have been informed of your participation in the study.**

Please take as much time as you need to decide and then let your doctor know what you have decided so that your treatment can be arranged.

**Thank you for taking time to read this leaflet and considering taking part in this study. You may use this information sheet to make notes or write down any questions you may have.**
