# Supplementary material for: Investigator choice of standard therapy versus sequential novel therapy arms in the treatment of relapsed follicular lymphoma (REFRACT): study protocol for a multi-centre, open-label, randomised, phase II platform trial
Source: BMC Cancer. 2024 Mar 25;24:370. doi: 10.1186/s12885-024-12112-0 (PMC10962099; doi:10.1186/s12885-024-12112-0)
Supplement: Supplementary file 6 — Supplementary Material 6 [file 12885_2024_12112_MOESM6_ESM.docx]

| Supplementary Appendix 5: REFRACT Schedule of Events  **Standard of care arm: Investigator choice therapy** | | | | | | | | | | | | | | |
| --- | --- | --- | --- | --- | --- | --- | --- | --- | --- | --- | --- | --- | --- | --- |
| **Assessment** | **Screening** (28 days)**^1^** | | **Treatment Phase** | | | | | | | | | **Follow up post end of induction treatment^3^** | | Annual follow up after treatment for survival, progression, subsequent treatment and any significant treatment-related adverse events |
|  |  |  | **C1 D1** | **C2 D1** | **C3 D1** | **C4 D1** | **C5 D1** | **C6 D1** | **End of induction treatment^2^** | **24 weeks from treatment start^2^** | | **60 days** | **24 weekly** |  |
| Informed consent | x | |  |  |  |  |  |  |  |  | |  |  |  |
| Demographic data, medical history | x | |  |  |  |  |  |  |  |  | |  |  |  |
| Confirmation of eligibility | x | |  |  |  |  |  |  |  |  | |  |  |  |
| Vital signs including temperature, blood pressure, pulse, respiration rate and oxygen saturation^4^ | x | | x | x | x | x | x | x | x |  | |  |  |  |
| Physical examination including lymph node exam^4^ | x | | x | x | x | x | x | x | x |  | |  |  |  |
| Height, weight and BSA (if applicable)^4,5^ | x | | x | x | x | x | x | x | x |  | |  |  |  |
| ECG | x | |  | As clinically indicated | | | | | | | |  |  |  |
| ECOG Performance Status^4^ | x | | x | x | x | x | x | x | x |  | |  |  |  |
| Pregnancy test^4,6^ | x^1^ | | x | x | x | x | x | x | x |  | |  |  |  |
| **Blood tests** |  |  | | | | | | | | | | | |  |
| FBC, biochemistry, LDH^4,7^ | x | | x | x | x | x | x | x | x | x | | x |  |  |
| Coagulation | x | | As clinically indicated | | | | | |  |  | |  |  |  |
| Virology (HIV, Hep B & C), CMV serology and PCR^8^ | x | |  |  |  |  |  |  |  |  | |  |  |  |
| Immunoglobulins (IgA, IgG and IgM) | x | |  |  |  |  |  |  | x^4^ |  | |  |  |  |
| Urinalysis | x | | As clinically indicated | | | | | |  |  | |  |  |  |
| PET-CT scan^9,10^ | x^1^ | |  |  | x^11^ |  |  |  |  | x^12^ | |  |  |  |
| **Samples** |  | | | | | | | | | | | | |  |
| Tumour biopsy^9^ | x^1^ | |  |  | x^13^ |  |  |  |  |  | |  |  |  |
| Bone marrow biopsy/aspirate (if clinically indicated)^9^ | x | | As clinically indicated and at disease progression | | | | | | | | | | |  |
| Research blood samples^9^ |  | | x |  |  | x |  |  | x^14^ | x^14^ | |  | X^15^ |  |
| Saliva | x | |  |  |  |  |  |  |  |  | |  |  |  |
| Quality of Life Questionnaires (EQ-5D-5L and FACT-Lym))^4,16^ | x | |  |  | x |  |  |  |  | x |  |  | x |  |
| Premedication^17^ |  | | x | x | x | x | x | x |  |  | |  |  |  |
| Treatment Administration^18^ |  | | x | x | x | x | x | x |  |  | |  |  |  |
| Concomitant Medications | Continuous assessment  Continuous assessment | | | | | | | | | | | |  |  |
| Adverse Events^19^ | Continuous assessment | | | | | | | | | | | | |  |

1. Screening assessments to be carried out within 28 days of trial entry with the exception of the following:
   1. A. tumour biopsy can be taken within 6 months of trial entry
   2. B. PET/CT may be performed within 6 weeks prior totrial entry
   3. C. pregnancy test must be taken within 7 days of treatment start
2. Note that these time points may coincide depending on the ICT
3. Patients will be followed up at 60 days and then every 6 months following treatment discontinuation or completion
4. Can be performed within 7 days. Vital signs and physical exam are acceptable within 7 days if the patient is well; otherwise repeat on the day of dosing.
5. Height to be taken at screening only. Body surface area (BSA) (if applicable) to be calculated as per local practice and to be recalculated if necessary depending on weight changes as per local practice (i.e. following a 10% change in weight)
6. A pregnancy test must be taken in women of child-bearing potential within 7 days of trial entry. Subsequent pregnancy tests must be taken every 4 weeks (before the start of each cycle) during treatment and once post treatment in women of child-bearing potential taking lenalidomide
7. FBC (to include haemoglobin, platelets, white blood cells, neutrophils, lymphocytes) and biochemistry to include (albumin, direct bilirubin, AST/ALT, ALP, calcium, creatinine, c-reactive protein, LDH, potassium, phosphate, uric acid (or urate), GFR, urea, total protein, sodium).
8. If patient is Hep B DNA positive they must be monitored as per local practice. For patients who become HBsAg or anti-HBc positive and HBV-DNA positive, treatment with anti-viral medication should be considered, per local practice
9. Also to be performed at disease progression
10. PET-CT with low dose CT are required; contrast enhanced CT scans are not required
11. *12 week PET/CT for research is optional. In the case of treatment delays the 12 week scan should be moved accordingly, to take place 4-6 weeks after cycle 3 day 1 and before cycle 4 day 1.

- **Please note for R-CHOP and R-CVP the 12 week scan would be a 9 week scan 3-5 weeks after cycle 3 day 1 and before cycle 4 day 1.*

1. The scan should be timed at 24 weeks from day 1 of cycle 1 of treatment. A +/- 2 week window is permitted, however scans must be performed a minimum of 4 weeks from end of treatment to avoid treatment related inflammation. In the case of treatment delays the 24-week scan should be moved accordingly, to take place 4-6 weeks after the end of treatment.
2. Cycle 2 day 15 (-/+ 3 days) biopsy for research is optional
3. Research blood samples to be taken at 24 weeks and end of treatment. Note if these time points coincide only 1 set of samples is required
4. Samples to be collected at 12 months following treatment completion
5. Quality of life questionnaires will be collected at screening, day 1 of cycle 3, at 24 weeks and then every 24 weeks in non-progressed patients until the end of study
6. Premedication is recommended with rituximab and obinutuzumab (see section 7.5.5.1 and 7.5.5.2 for further information)
7. Treatment schedules will vary depending on the ICT, please see section 7.3 for recommended treatment schedules including optional rituximab/obinutuzumab maintenance
8. Adverse events will be collected for 12 months (+ 60 days)

| **Experimental arm round 1: epcoritamab and lenalidomide** | | | | | | | | | | | | | | |
| --- | --- | --- | --- | --- | --- | --- | --- | --- | --- | --- | --- | --- | --- | --- |
| **Assessment** | **Screening**  (28 days)**^1^** | **Treatment Phase** | | | | | | | | | | **FU post end of treatment^3^** | | Annual follow up after treatment for survival, progression, subsequent treatment and any significant treatment-related adverse events |
|  |  | **Cycle 1-3^18^** | | | | | | | **Cycle 4-12** | **24 weeks from treatment start^2^** | **End of Treatment** | **60 days** | **24 weekly** |  |
|  |  | D1  D8 | | D8^18^  D22 | | D15 | D22^18^ | | D1 |  |  |  |  |  |
| Informed consent | x |  | |  | |  |  | |  |  |  |  |  |  |
| Demographic data, medical history | x |  | |  | |  |  | |  |  |  |  |  |  |
| Confirmation of eligibility | x |  | |  | |  |  | |  |  |  |  |  |  |
| Vital signs including temperature, blood pressure, pulse, respiration rate and oxygen saturation^4^ | x | x | | x | | x | x | | x |  | x |  |  |  |
| Physical examination including lymph node exam^4^ | x | x | | x | | x | x | | x |  | x |  |  |  |
| Neurology/neurotoxicity Assessment^5^ |  | x | |  | | x^4^ |  | | x^4^ |  | x^4^ |  |  |  |
| Height, weight and BSA (if applicable)^4,6^ | x | x | |  | |  |  |  | x |  | x |  |  |  |
| ECG | x |  | | | As clinically indicated | | | | | | |  |  |  |
| ECOG Performance Status^4^ | x | x | |  | |  |  | | x |  | x |  |  |  |
| Pregnancy test^4,7^ | x^1^ | x | |  | |  |  | | x |  | x |  |  |  |
| **Blood tests** |  | |  | | | | | | | | | | |  |
| FBC, biochemistry, LDH^4,8^ | x | x | | x | | x | x | | x | x | x | x |  |  |
| Coagulation | x | As clinically indicated | | | | | | | |  |  |  |  |  |
| Virology (HIV, Hep B & C), CMV serology and PCR^9^ | x |  | |  | |  |  | |  |  |  |  |  |  |
| Immunoglobulins (IgA, IgG and IgM) | x |  | |  | |  |  | |  |  | x^4^ |  |  |  |
| Urinalysis | x | As clinically indicated | | | | | | | |  |  |  |  |  |
| PET-CT scan^10,11^ | x^1^ |  | |  | |  |  | | x^12^ | x^13^ |  |  |  |  |
| **Samples** |  | | | | | | | | | | | | |  |
| Tumour biopsy^10^ | x^1^ |  | |  | | x^14^ |  | |  |  |  |  |  |  |
| Bone marrow biopsy/aspirate (if clinically indicated)^10^ | x | As clinically indicated and at disease progression | | | | | | | | | | | |  |
| Research blood samples^10^ | x |  | |  | |  |  | | x (cycle 4 day 1) | x | x |  | X^15^ |  |
| Saliva | x |  | |  | |  |  | |  |  |  |  |  |  |
| Quality of Life Questionnaires (EQ-5D-5L and FACT-Lym))^4,16^ | x | x (cycle 3) | |  | |  |  | |  | x |  |  | x |  |
| CRS Prophylaxis^17^ |  | x | | x | | x | x | | |  |  |  |  |  |
| Epcoritamab Administration |  | x | | x | | x | x | | x |  |  |  |  |  |
| Lenalidomide Administration |  |  | | | Continuous 20mg od days 1-21 | | | | | |  |  |  |  |
| Concomitant Medications | Continuous assessment | | | | | | | | | | | |  |  |
| Adverse Events | Continuous assessment | | | | | | | | | | | |  |  |

1. Screening assessments to be carried out within 28 days of trial entry with the exception of the following:
   1. A. tumour biopsy can be taken within 6 months of trial entry
   2. B. PET/CT may be performed within 6 weeks prior to trial entry
   3. C. pregnancy test must be taken within 7 days of treatment start
2. For patients on the experimental arm cycle 7 day 1 assessments may be used if the patient is continuing treatment
3. Patients will be followed up at 60 days and then every 6 months following treatment discontinuation or completion
4. Can be performed within 7 days. Vital signs and physical exam are acceptable within 7 days if the patient is well; otherwise repeat on the day of dosing.
5. Neurology assessment to be performed at the time points above and additionally whenever clinically indicated
6. Height to be taken at screening only. Body surface area (BSA) (if applicable) to be calculated as per local practice and to be recalculated if necessary depending on weight changes as per local practice (i.e. following a 10% change in weight)
7. A pregnancy test must be taken in women of child-bearing potential within 7 days of trial entry. Subsequent pregnancy tests must be taken every 4 weeks (before the start of each cycle) during treatment and once post treatment in women of child-bearing potential taking lenalidomide
8. Full blood count (to include haemoglobin, platelets, white blood cells, neutrophils, lymphocytes) and biochemistry to include (albumin, direct bilirubin, AST/ALT, ALP, calcium, creatinine, c-reactive protein, LDH, potassium, phosphate, uric acid (or urate), GFR, urea, total protein, sodium).
9. If patient is Hep B DNA positive they must be monitored as per local practice. For patients who become HbsAg or anti-HBc positive and HBV-DNA positive, treatment with anti-viral medication should be considered, per local practice
10. Also to be performed at disease progression
11. PET with low dose CT are required; contrast enhanced CT scans are not required
12. 12 week PET/CT for research is optional. In the case of treatment delays the 12 week scan should be moved accordingly, to take place 4-6 weeks after cycle 3 day 1 and before cycle 4 day 1.
13. Scan can be performed at 24 weeks (a 2 week window is permitted, but must be no earlier than 24 weeks). In the case of treatment delays the 24-week scan should be moved accordingly, to take place 4-6 weeks after cycle 6 day 1.
14. Cycle 2 day 15 (-/+ 3 weeks) biopsy for research is optional
15. Samples to be collected at 12 months following treatment completion
16. Quality of life questionnaires will be collected at screening, day 1 of cycle 3, at 24 weeks and then every 24 weeks in non-progressed patients until the end of study
17. CRS prophylaxis with corticosteroids is mandated during cycle 1 and may be continued beyond cycle 1 as required (see section 7.5.5.3 for further information)
18. For Cycle 3, D8 and D15 assessments may be omitted if there were no complications during previous cycles (see section 8 for further information)
