# Supplementary material for: Investigator choice of standard therapy versus sequential novel therapy arms in the treatment of relapsed follicular lymphoma (REFRACT): study protocol for a multi-centre, open-label, randomised, phase II platform trial
Source: BMC Cancer. 2024 Mar 25;24:370. doi: 10.1186/s12885-024-12112-0 (PMC10962099; doi:10.1186/s12885-024-12112-0)
Supplement: Supplementary file 7 — Supplementary Material 7 [file 12885_2024_12112_MOESM7_ESM.docx]

# Supplementary Appendix 6: Recommended treatment schedules for the investigator choice of standard therapy (ICT)

Note: Infusion rates of each drug should follow local protocols. Dose calculations for patients with a body surface area >2.2m^2^ should be according to local policies.

Rituximab and bendamustine

The recommended starting dose of rituximab is 375 mg/m^2^ intravenously (IV) every week in Cycle 1 (days 1, 8, 15, and 22) and day 1 of every 28-day cycle for six cycles. Bendamustine should be administered at a dose of 90mg/m^2^ on days 1 and 2 of each cycle for six cycles.

Patients who achieved a complete or partial response (at the Investigator’s discretion) following the initial six treatment cycles or have stable disease could continue to receive rituximab 375/mg/m^2^ as single agent maintenance therapy once every two months for two years or until disease progression (whichever occurs first).

Rituximab and cyclophosphamide, vincristine, and prednisolone (CVP)

Patients will be treated with rituximab-CVP for six 21-day cycles according to the following schedule:

Table S61: Rituximab-CVP schedule

| **Drug** | **Dose^*^** | **Route of administration** | **Days of cycle** |
| --- | --- | --- | --- |
| Rituximab | 375 mg/m^2^ | IV infusion | 1 |
| Cyclophosphamide | 750 mg/m^2^ | IV bolus/infusion | 1 |
| Vincristine* | 1.4 mg/m^2^ | IV infusion | 1 |
| Prednisolone | 100mg or 40mg/m^2^ | PO | 1-5 |

*Note maximum dose of vincristine is 2 mg, or 1 mg in patients >70 years

Patients who achieved a complete or partial response (at the Investigator’s discretion) following the initial six treatment cycles or have stable disease could continue to receive rituximab 375/mg/m^2^ as single agent maintenance therapy once every two months for two years or until disease progression (whichever occurs first).

Rituximab and cyclophosphamide, doxorubicin, vincristine, and prednisolone (CHOP)

Patients will be treated with rituximab-CHOP for six 21-day cycles according to the following schedule:

Table S62: Rituximab-CHOP schedule

| **Drug** | **Dose^*^** | **Route of administration** | **Days of cycle** |
| --- | --- | --- | --- |
| Rituximab | 375 mg/m^2^ | IV infusion | 1 |
| Cyclophosphamide | 750 mg/m^2^ | IV bolus/infusion | 1 |
| Vincristine* | 1.4 mg/m^2^ | IV infusion | 1 |
| Prednisolone | 100mg or 40mg/m^2^ | PO | 1-5 |

*Note maximum dose of vincristine is 2 mg, or 1 mg in patients >70 years

Patients who achieved a complete or partial response (at the Investigator’s discretion) following the initial six treatment cycles or have stable disease could continue to receive rituximab 375/mg/m^2^ as single agent maintenance therapy once every two months for two years or until disease progression (whichever occurs first).

Rituximab and lenalidomide

The recommended starting dose of lenalidomide is 20 mg, orally once daily on days 1 to 21 of repeated 28-day cycles for up to 12 cycles of treatment. The recommended starting dose of rituximab is 375 mg/m^2^ IV every week in Cycle 1 (days 1, 8, 15, and 22) and day 1 of every 28-day cycle for cycles 2 through 5.

See Dose Modifications and Discontinuations section of the main text for cycle starting criteria related to lenalidomide.

Obinutuzumab and bendamustine

Obinutuzumab should be administered in six 28-day cycles in combination with bendamustine.

Patients who achieved a complete or partial response (at the Investigator’s discretion) following the initial six treatment cycles or have stable disease could continue to receive obinutuzumab 1,000 mg as single agent maintenance therapy once every two months for two years or until disease progression (whichever occurs first).

Table S63: Obinutuzumab and bendamustine schedule

| **Cycle** | **Day of treatment** | **Obinutuzumab** | **Bendamustine** |
| --- | --- | --- | --- |
| Cycle 1 | Day 1* | 1,000 mg | 90 mg/m^2^ |
|  | Day 2* | n/a | 90 mg/m^2^ |
|  | Day 8 | 1,000 mg | n/a |
|  | Day 15 | 1,000 mg | n/a |
| Cycles 2–6 | Day 1 | 1,000 mg | 90 mg/m^2^ |
|  | Day 2 | n/a | 90 mg/m^2^ |
| Maintenance | Every 2 months for 2 years or until disease progression (whichever occurs first) | 1,000 mg | n/a |

*Split dose of obinutuzumab on days 1 and 2 of cycle 1 is permitted, as per local practice.

If a planned dose of obinutuzumab is missed, it should be administered as soon as possible; do not omit it or wait until the next planned dose. If toxicity occurs before Cycle 1 Day 8 or Cycle 1 Day 15, requiring delay of treatment, these doses should be given after resolution of toxicity. In such instances, all subsequent visits and the start of Cycle 2 will be shifted to accommodate for the delay in Cycle 1 with infusion rates of each drug following local protocols.
