# Supplementary material for: Investigator choice of standard therapy versus sequential novel therapy arms in the treatment of relapsed follicular lymphoma (REFRACT): study protocol for a multi-centre, open-label, randomised, phase II platform trial
Source: BMC Cancer. 2024 Mar 25;24:370. doi: 10.1186/s12885-024-12112-0 (PMC10962099; doi:10.1186/s12885-024-12112-0)
Supplement: Supplementary file 8 — Supplementary Material 8 [file 12885_2024_12112_MOESM8_ESM.docx]

# **Supplementary Appendix 7: Recommended dose modifications for the permitted investigator choice of standard therapies**

## Rituximab or obinutuzumab

There are no specific dose modifications for rituximab or obinutuzumab, dose modifications may be made as per local practice.

## Cyclophosphamide, vincristine, and prednisolone (CVP) and cyclophosphamide, doxorubicin, vincristine, and prednisolone (CHOP) dose reductions for haematological toxicity

| **Toxicity** | **Dose Reduction** |
| --- | --- |
| **Cyclophosphamide** | |
| Leukocyte count (x10^9^/L)  25-40  <25 | 50%  Omit until values normalise or decide individually |
| Platelet count (x10^9^/L)  50-100  <50 | 50%  Omit until values normalise or decide individually |
| **Doxorubicin** | |
| Platelet count (x10^9^/L)  50-74 | Consider 25% |
| **All doses** | |
| Neutrophils (x10^9^/L)  0.5-<1.0  <0.5 | If patient is fit and well, proceed with treatment and give G-CSF from Day 6 if not already prescribed. If patient is unwell, delay for 1 week  Consider delay by one week |
| Platelets (x10^9^/L)  <50 | Consider delay by one week |

## Cyclophosphamide, vincristine, and prednisolone (CVP) and cyclophosphamide, doxorubicin, vincristine, and prednisolone (CHOP) dose reductions for non-haematological toxicity

| **Toxicity** | **Dose Reduction** |
| --- | --- |
| **Cyclophosphamide** | |
| Renal Impairment  GFR < 10 ml/min | 50% |
| Hepatic Impairment  Bilirubin 53 – 86 mmol/L | 25% |
| **Vincristine** | |
| Hepatic Impairment  Bilirubin > 51 mmol/L | 50% |
| **Doxorubicin (CHOP only)** | |
| Renal Impairment  GFR < 10 ml/min | 25% |
| Hepatic Impairment  Bilirubin 20-50 mmol/L  Bilirubin > 50 mmol/L | 50%  75% |

## Bendamustine

For haematological toxicity consider terminating or delaying treatment if leukocytes <3 x10^9^/L or platelets <75 x10^9^/L Treatment may be resumed if leukocytes >4 x10^9^/L and platelets >100 x10^9^/L

For non-haematological toxicity a 50% dose reduction is recommended in the case of a grade 3 toxicity. An interruption of treatment is recommended in case of grade 4 toxicity.
