# Supplementary material for: Investigator choice of standard therapy versus sequential novel therapy arms in the treatment of relapsed follicular lymphoma (REFRACT): study protocol for a multi-centre, open-label, randomised, phase II platform trial
Source: BMC Cancer. 2024 Mar 25;24:370. doi: 10.1186/s12885-024-12112-0 (PMC10962099; doi:10.1186/s12885-024-12112-0)
Supplement: Supplementary file 9 — Supplementary Material 9 [file 12885_2024_12112_MOESM9_ESM.docx]

# **Supplementary Appendix 8: Pre-medication to prevent infusion-related reactions**

## Rituximab

For patients on rituximab in any treatment combination, pre-medication before each rituximab infusion must be given prior to the treatment as follows (or as per local practice):

| **Anti-pyretic e.g. paracetamol** | **Antihistamine e.g., chlorphenamine** | **Glucocorticoid**  **(If not being given in combination with a glucocorticoid-containing chemotherapy)** |
| --- | --- | --- |
| 1000 mg PO | 10 mg PO/IV | Hydrocortisone 100mg IV  Note for CHOP/CVP: Day 1 treatment dose of oral prednisolone may be used as glucocorticoid pre-medication prior to rituximab infusion. |

CHOP, Rituximab and cyclophosphamide, doxorubicin, vincristine, and prednisolone; CVP, Rituximab and cyclophosphamide, vincristine, and prednisolone; IV, intravenous; PO, oral.

## Obinutuzumab

Corticosteroid pre-medication is recommended for patients receiving obinutuzumab in their first cycle. Pre-medication for subsequent infusions and other pre-medication should be administered as described in the table below.

Hypotension, as a symptom of infusion-related reactions (IRRs) may occur during obinutuzumab intravenous infusions. Therefore, withholding of antihypertensive treatments should be considered for 11 hours prior to and throughout each infusion and for the first hour after administration.

| **Day of treatment cycle** | **Patients requiring premedication** | **Premedication** | **Administration** |
| --- | --- | --- | --- |
| **Cycle 1 day 1** | All patients | Intravenous corticosteroid^1^  (recommended) | Completed at least 1 hour prior to obinutuzumab infusion |
|  |  | Oral analgesic/anti-pyretic^2^ | At least 30 minutes before infusion |
|  |  | Anti-histaminic medicine^3^ |  |
| **All subsequent infusions** | Patients with no IRR during the previous infusion | Oral analgesic/anti-pyretic^2^ | At least 30 minutes before infusion |
|  | Patients with an IRR (grade 1 or 2) with the previous infusion | Oral analgesic/anti-pyretic^2^  Anti-histaminic medicine^3^ |  |
|  | Patients with a grade 3 IRR with the previous infusion OR lymphocyte >25x10^9^/L prior to next treatment | Intravenous corticosteroid^1^ | At least 1 hour before infusion |
|  |  | Oral analgesic/anti-pyretic^2^  Anti-histaminic medicine^3^ | At least 30 minutes before infusion |

^1^ 100 mg prednisolone or 20 mg dexamethasone or 80 mg methylprednisolone. Hydrocortisone should not be used as it has not been effective in reducing rates of infusion-related reaction (IRR).

^2^ e.g., 1,000 mg paracetamol

^3^ e.g., 10 mg chlorphenamine
