# Supplementary material for: Investigator choice of standard therapy versus sequential novel therapy arms in the treatment of relapsed follicular lymphoma (REFRACT): study protocol for a multi-centre, open-label, randomised, phase II platform trial
Source: BMC Cancer. 2024 Mar 25;24:370. doi: 10.1186/s12885-024-12112-0 (PMC10962099; doi:10.1186/s12885-024-12112-0)
Supplement: Supplementary file 10 — Supplementary Material 10 [file 12885_2024_12112_MOESM10_ESM.docx]

# Supplementary Appendix 9: Concomitant medications for patients receiving investigator choice of standard therapy

## Bendamustine

The combination of bendamustine with cyclosporine or tacrolimus may result in excessive immunosuppression with risk of lymphoproliferation. In addition, bendamustine metabolism involves cytochrome P450 (CYP) 1A2 isoenzyme. Therefore, the potential for interaction with CYP1A2 inhibitors exists (see Tables S9A-C).

### Table S9A: CYP1A2 Inhibitors

| Abiraterone | Enoxacin | Quinidine |
| --- | --- | --- |
| Amiodarone | Fluvoxamine | Rofecoxib |
| Ciprofloxacin | Furafylline | Technetium Tc-99m ciprofloxacin |
| Clinafloxacin | Midostaurin | Zafirlukast |

### Table S9B: Moderate CYP1A2 Inhibitors

| Alosetron | Gatifloxacin | Lobeglitazone | Pazufloxacin |
| --- | --- | --- | --- |
| Bortezomib | Imipramine | Mexiletine | Simeprevir |
| Caffeine | Ketoconazole | Moxifloxacin | Vemurafenib |
| Curcumin | Lidocaine | Osilodrostat | Zucapsaicin |
| Dosulepin |  |  |  |

### Table S9C: Weak CYP1A2 Inhibitors

| Anagrelide | Estradiol dienanthate | Pipemidic acid |
| --- | --- | --- |
| Cimetidine | Estradiol valerate | Quercetin |
| Citalopram | Ethambutol | Rucaparib |
| Clascoterone | Famotidine | Tirbanibulin |
| Conjugated estrogens | Genistein | Tocainide |
| Estradiol | Mefenamic acid | Trovafloxacin |
| Estradiol acetate | Nevirapine | Viloxazine |
| Estradiol benzoate | Opicapone |  |

## Cyclophosphamide, vincristine, and prednisolone (CVP) and cyclophosphamide, doxorubicin, vincristine, and prednisolone (CHOP)

The following medications should be used with caution in patients receiving CHOP or CVP:

- Strong/moderate inducers of CYP3A (see Tables S9D and E)
- Strong/moderate inhibitors of CYP3A (see Tables S9F and G)
- L-asparaginase
- Isoniazid (and other drugs acting on the nervous system)
- Phenobarbital, phenytoin, carbamazepine
- NSAIDs
- Oestrogen’s
- Potassium reducing agents
- Rifampicin
- Anticholinergic neuromuscular blockers such as pancuronium and vecuronium
- Anticholinesterases such as ambenonium, neostigmine and pyridostigmine

The following medications should be used with caution in patients receiving doxorubicin (CHOP only):

- Amphotericin B
- Inhibitors of P-glycoprotein (see Table S9H)
- Clozapine

### Table S9D: Strong CYP3A Inducers

| Apalutamide | Fosphenytoin | Phenobarbital | Rifapentine |
| --- | --- | --- | --- |
| Carbamazepine | Lumacaftor | Phenytoin | Rifaximin |
| Clotrimazole | Midostaurin | Primidone | Rimexolone |
| Dexamethasone | Mitotane | Rifampicin | St. John's Wort |
| Enzalutamide | Pentobarbital | Rifamycin |  |

### Table S9E: Moderate CYP3A Inducers

| Avasimibe | Dexamethasone acetate | Etravirine |
| --- | --- | --- |
| Bexarotene | Echinacea | Modafinil |
| Bosentan | Efavirenz | Nafcillin |
| Dexamethasone |  |  |

### Table S9F: Strong CYP3A Inhibitors

| Amiodarone | Delavirdine | Levoketoconazole | Ribociclib |
| --- | --- | --- | --- |
| Amprenavir | Diltiazem | Lonafarnib | Ritonavir |
| Atazanavir | Ditiocarb | Loperamide | Saquinavir |
| Boceprevir | Econazole | Lopinavir | Stiripentol |
| Clarithromycin | Efavirenz | Methimazole | Telaprevir |
| Cobicistat | Elvitegravir | Midostaurin | Telithromycin |
| Conivaptan | Ergotamine | Naloxone | Terfenadine |
| Curcumin | Idelalisib | Nefazodone | Tipranavir |
| Danazol | Indinavir | Nelfinavir | Troleandomycin |
| Danoprevir | Itraconazole | Nilotinib | Voriconazole |
| Darunavir | Ketoconazole |  |  |

### Table S9G: Moderate CYP3A Inhibitors

| Abiraterone | Desvenlafaxine | Isavuconazole | Nilvadipine |
| --- | --- | --- | --- |
| Aprepitant | Diltiazem | Isavuconazonium | Primaquine |
| Barnidipine | Dronedarone | Isoniazid | Seproxetine |
| Benidipine | Erythromycin | Isradipine | Simeprevir |
| Berotralstat | Fluconazole | Linagliptin | Tioconazole |
| Ciprofloxacin | Fluvoxamine | Lovastatin | Venetoclax |
| Clindamycin | Fosamprenavir | Luliconazole | Verapamil |
| Clozapine | Fosnetupitant | Miconazole | Voriconazole |
| Crizotinib | Fusidic acid | Milnacipran | Zimelidine |
| Cyclosporine | Haloperidol | Netupitant | Ziprasidone |
| Danazol | Indalpine | Nicardipine |  |

### Table S9H: Pgp Inhibitors

| Acetaminophen | Citalopram | Fluconazole | Mefloquine | Promethazine |
| --- | --- | --- | --- | --- |
| Alfentanil | Clarithromycin | Fluoxetine | Megestrol acetate | Propranolol |
| Amiodarone | Clomipramine | Flupentixol | Methadone | Quinine |
| Amlodipine | Clotrimazole | Fluvoxamine | Methylene blue | Ranitidine |
| Atorvastatin | Cyclosporine | Galantamine | Metronidazole | Sertraline |
| Atovaquone | Daunorubicin | Haloperidol | Miconazole | Sildenafil |
| Azithromycin | Dexamethasone | Hydroxychloroquine | Mifepristone | Simvastatin |
| Bisoprolol | Digoxin | Ibuprofen | Mirabegron | Sirolimus |
| Bromocriptine | Diltiazem | Indomethacin | Naproxen | Tacrolimus |
| Buprenorphine | Dipyridamole | Isavuconazole | Netupitant | Telmisartan |
| Buspirone | Doxazosin | Itraconazole | Nicardipine | Temsirolimus |
| Canagliflozin | Doxorubicin | Ivermectin | Nifedipine | Tenofovir disoproxil |
| Candesartan | Dronedarone | Ketoconazole | Norethisterone | Testosterone |
| Cannabidiol | Duloxetine | Lamotrigine | Omeprazole | Ticagrelor |
| Cannabinol | Enalapril | Lansoprazole | Pantoprazole | Tolvaptan |
| Captopril | Ergotamine | Letermovir | Paroxetine | Trimethoprim |
| Carvedilol | Erythromycin | Levofloxacin | Polyethylene glycol | Venetoclax |
| Caspofungin | Esomeprazole | Lidocaine | Posaconazole | Venlafaxine |
| Ceftriaxone | Everolimus | Linagliptin | Prazosin | Verapamil |
| Cetirizine | Felodipine | Loratadine | Prednisone | Vinblastine |
| Chloroquine | Fenofibrate | Losartan | Primaquine | Vincristine |
| Chlorpromazine | Fentanyl | Medroxyprogesterone acetate | Progesterone |  |
