# Supplementary material for: Investigator choice of standard therapy versus sequential novel therapy arms in the treatment of relapsed follicular lymphoma (REFRACT): study protocol for a multi-centre, open-label, randomised, phase II platform trial
Source: BMC Cancer. 2024 Mar 25;24:370. doi: 10.1186/s12885-024-12112-0 (PMC10962099; doi:10.1186/s12885-024-12112-0)
Supplement: Supplementary file 11 — Supplementary Material 11 [file 12885_2024_12112_MOESM11_ESM.pdf]

Statistical Analysis Plan

Relapsed Follicular lymphoma Randomised trial Against standard ChemoTherapy (REFRACT): A randomised phase II trial of investigator choice standard therapy versus sequential novel therapy experimental arms

Version: 2.0

Date: 12-September-2023

**Sponsor:**	University of Birmingham
**Sponsor reference number**	RG_22-020
**CRCTU reference number**	HM2072
**EudraCT number**	2022-000677-75
**Clinicaltrials.gov**	NCT05848765

KEY PERSONNEL INVOLVED IN THE PREPARATION OF THE STATISTICAL ANALYSIS PLAN:

NAME	TRIAL ROLE
Charlotte Gaskell	Trial Biostatistician
Aimee Jackson	Lead Biostatistician
Dr Kim Linton	Deputy Chief Investigator
Dr Mark Bishton	Chief Investigator

DOCUMENT CONTROL SHEET

STATISTICAL ANALYSIS PLAN VERSION:	REASON FOR UPDATE:
V1.0 (01-Sep-2022)	First version of the statistical analysis plan written in conjunction with version 1.0 of the protocol
V2.0 (12-Sep-2023)	Minor grammatical updates throughout and the following updated in line with protocol v3.0:<br>• Addition of clinicaltrials.gov number to front page<br>• Updates to exploratory outcome wording (Section 1.2)<br>• Addition of central PET review and how this will be incorporated into the analysis (Section 8.1.1)

1. INTRODUCTION

1.1 PURPOSE OF THE STATISTICAL ANALYSIS PLAN

This Statistical Analysis Plan (SAP) provides guidelines for the analysis and presentation of results for the REFRACT trial. This plan, along with all other documents relating to the analysis of this trial, will be stored in the 'Statistical Documentation' section of the Trial Master File. The statistical analysis will be carried out by the Trial Statistician.

1.2 SUMMARY OF THE TRIAL

Trial Design

REFRACT is a prospective, randomised, phase II platform trial for sequential evaluation of experimental treatments versus investigator choice standard therapy (ICT) for patients with relapsed and refractory Follicular Lymphoma (rrFL).

Objectives

<u>Primary</u>

To identify novel therapies with superior efficacy compared to ICT based on post-induction complete metabolic response (CMR) rate

<u>Secondary</u>

- To evaluate treatment response and survival of patients with rrFL treated with novel therapies.
- To identify novel therapies with high efficacy in high-risk FL
- To identify novel therapies with other advantages over standard treatments, such as safety and quality of life
- To evaluate clinical outcomes for ICT to provide new benchmarks.

<u>Exploratory</u>

- To explore the prognostic value of PET-CT radiomic features including PET-CT total metabolic tumour volume outcome measures
- To characterise and evaluate the predictive value of dynamic changes in the tumour microenvironment (TME) using deconvoluted bulk cell RNA sequencing.
- To evaluate the predictive and prognostic value of baseline, interim and end of treatment ctDNA levels and the peripheral blood immune composition
- To classify TME classes using imaging mass cytometry, identify predictive and treatment guiding biomarkers and new druggable targets.

Outcome Measures

<u>Primary Endpoint</u>

Complete metabolic response (CMR) by PET-CT at 24 weeks using the Deauville 5-point scale and Lugano 2014 criteria (see Appendix 2 of the protocol).

Secondary Endpoints

- Overall metabolic response (CMR + partial metabolic response (PMR)) by PET-CT at 24 weeks.
- Progression free survival (PFS) defined as the time from randomisation to the date of first disease progression or death from any cause.
- Overall survival (OS) defined as time from randomisation to the date of death from any cause.
- Duration of response (DoR) defined as the time from complete and partial metabolic response to relapse/progression or death from any cause.
- Duration of complete response (DoCR) defined as the time from complete metabolic response to relapse/progression or death from any cause.
- Time to next treatment (TTNT) defined as the time from randomisation to the start date of next treatment for lymphoma. Patients who are responding (CMR or PMR) who receive consolidation radiotherapy will not be considered an event and will be censored at their date last seen if no other treatment for lymphoma is reported. Patients who die without having started next lymphoma treatment will be considered a competing risk at their date of death, and patients who are alive at the end of the trial and have not started next lymphoma treatment will be censored at their date last seen.
- Adverse events (AEs) collected and reported in accordance with CTCAE version 5 defined as the number of patients who experience one or more grade 3 or 4 adverse events or serious adverse events of any grade.
- Quality of life (QoL) (measured using the EQ-5D-5L, EQ-VAS and FACT-Lym) collected at pre-treatment, day 1 of cycle 3, week 24 and then every 24 weeks in non-progressed patients until the end of study (see Appendix 3 of the protocol).

Exploratory Endpoints

- Correlation of MTV with CMR, PFS, DOR and OS
- Frequency of immune cells in tumour samples computed from bulk RNA-sequencing data.
- Correlation of ctDNA changes with relapse biopsy, treatment response and disease progression.
- Peripheral blood immune cell composition, changes during treatment and correlation with treatment response
- TME classification by imagining mass cytometry and correlation with treatment response.

Patient Population and Sample Size

The study will recruit 284 patients with rrFL over 5 years: 95 control and 189 experimental arm patients.

Trial Duration

Patients will be recruited over a 5-year period. Patients will be followed up annually until the end of the study.

1.3 TRIAL SCHEMA

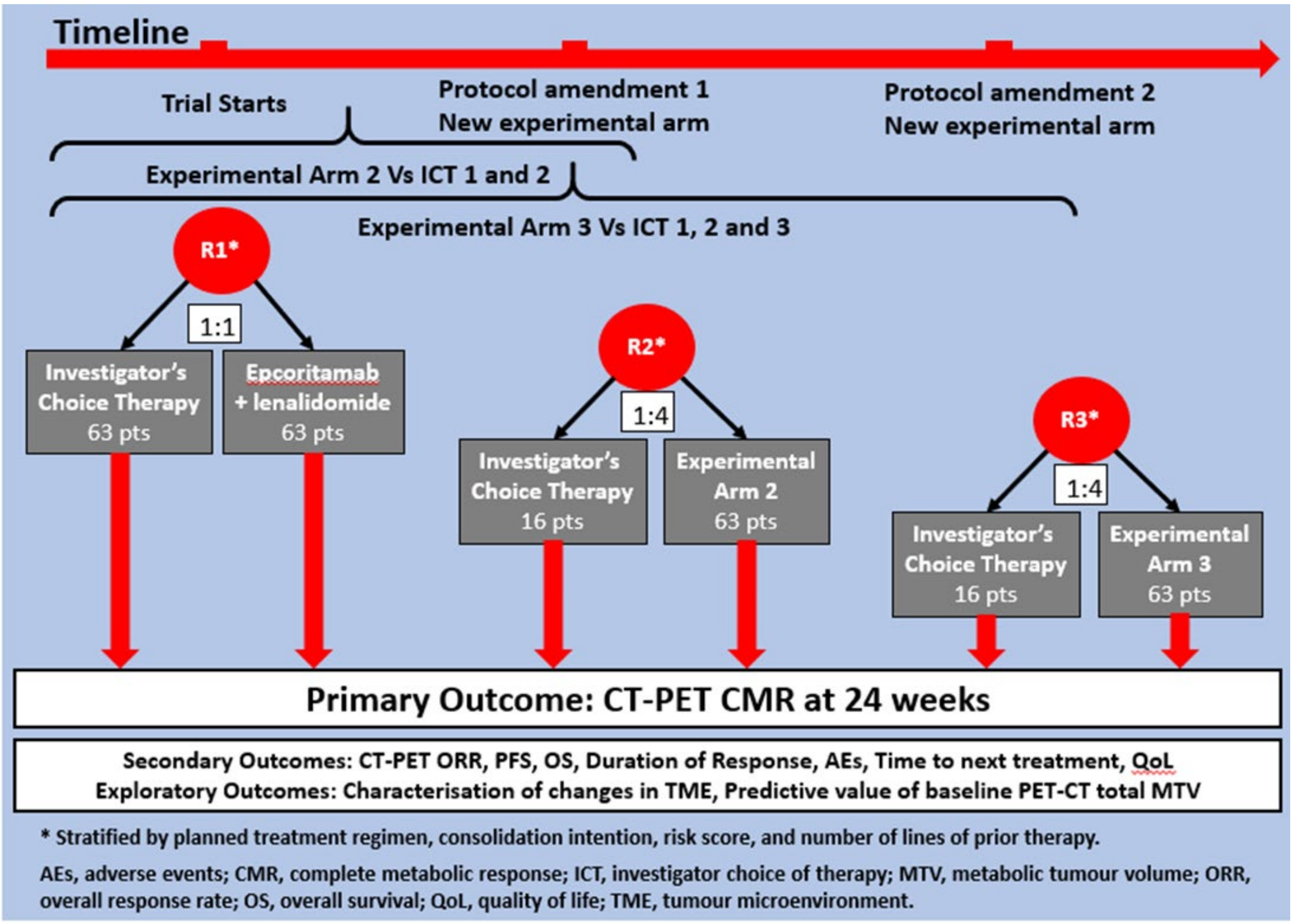

2. TIMING AND REPORTING OF INTERIM AND FINAL ANALYSES

2.1 INTERIM ANALYSES

Due to the limited size of the REFRACT trial, no formal statistical interim analyses have been defined within any of the three rounds. Accumulating data and analyses will be monitored regularly by an independent data monitoring committee (DMC) on a yearly basis, or more frequently if required. Following these reviews, the DMC will report to the Trial Management Group (TMG) who will in turn convey the findings of the DMC to the Trial Steering Committee (TSC) and the MHRA.

The DMC may consider recommending the discontinuation of the trial, or of a specific round within the trial, if the recruitment rate or data quality are unacceptable or if any issues are identified which may compromise patient safety. The trial, or specific rounds of the trial, may also stop early if the analyses presented to the DMC showed differences between treatments in favour of the experimental arm, that were deemed to be convincing to the clinical community.

2.2 MAIN ANALYSES

Given this trial is comprised of three separate rounds, and due to the short maturity time of the primary outcome, there are six planned main analysis timepoints. The relevant analyses have been defined below for R1, details regarding the main analyses of subsequent rounds will be updated once treatment comparison is known.

2.2.1 ROUND ONE: INVESTIGATOR CHOICE VS EPCORITMAB + LENALIDOMIDE

Main analysis for this treatment control comparison is planned to take place in two separate stages. The first analysis will be conducted when every patient within the comparison has reached 24 weeks follow-up and data for the primary endpoint has been received and cleaned. This analysis will comprise of analysis of the primary outcome, CMR by PET-CT at 24 weeks and any matured key secondary outcomes, anticipated to include Overall Metabolic Response at 24 weeks; Safety and Quality of Life.

Longer term endpoints for this treatment comparison will be analysed when all patients have a minimum of 3 years follow-up and data collection is complete. This analysis will include Progression Free Survival; Overall Survival; Duration of Response; Duration of Complete Response; Time-to-next-treatment and Quality of Life.

2.2.2 ROUND TWO

To be updated once treatment comparison is known.

2.2.3 ROUND THREE

To be updated once treatment comparison is known.

2.3 DATA MONITEERING COMMITTEE REPORTS

2.3.1 ANNUAL MEETING REPORTS

At each DMC meeting, two reports will be provided, an open and a closed report. Both reports are to be circulated by the trial statistician, however the open report should be written jointly with the trial co-ordinator (TC; items requiring input from the TC are marked with an asterisks). The open report will contain, at a minimum, the following information: a summary of the relevant trial information*; information pertaining to the trial status*; details of protocol* and SAP amendments; a summary of site set-up*; screening information including details of screen failures*; details of any serious breaches*; recruitment data*; baseline patient characteristics (not split by treatment arm); statement of withdrawals (not split by treatment arm); deviation details (not split by treatment arm); and follow-up information.

The closed report will be produced <u>only</u> by the trial statistician and will include, at a minimum, the following information, split by treatment arm where relevant: baseline patient characteristics; treatment compliance and discontinuations; withdrawals; deviations; primary outcome data (where mature and relevant); mature and relevant secondary outcome data (excluding quality of life) and safety data. Additional information requested by the DMC can be included within the closed report if it is deemed suitable and relevant.

2.3.2 WEIGHTING DECISION REPORTS – ROUNDS 2 AND 3 ONLY

In addition to the annual reports generated for the DMC, during Rounds 2 & 3 an additional report will be generated and circulated to all independent DMC members via email. This report will detail clearly to the DMC the preliminary decision made by the TMG regarding the weightings to be used within the primary analysis of the relevant round, justification of this decision and any relevant information required to assess this decision. The DMC will then review the decision made and reply via email, unless a meeting is required, as to whether they agree with the choice made. This will then be fed back to the TSC and a final decision regarding the weighting made.

This report will be generated once recruitment has completed for the relevant round and all baseline information that is pertinent to disease response has been collected and cleaned.

This report will be produced by the trial statistician in conjunction with the TC (*) and Chief Investigators (CIs; †) and will contain the following information, split by analysis round where relevant; dates of recruitment start and stop date for each round*, details of any changes to standard of care treatment in the time between the previous round's recruitment closing and the current round of recruitment closing†; tabulations of baseline characteristics, containing only those recruited to the control rounds, including but not limited to: the stratification factors used within the minimisation algorithm; age; sex; relevant haematological and biochemical assessments; ECOG performance status and PET-CT results at screening, the proposed weighting for the primary analysis, justification for the proposed weighting† and details of any proposed sensitivity analyses.

3. RECRUITMENT AND RANDOMISATION

3.1 RECRUITMENT

Recruitment information will be presented in all reports produced; this data will be summarised in the following ways:

- Date the relevant round opened and closed (where applicable) to recruitment.
- Date the snapshot used to generate the report was taken.
- The number of centres open to recruitment.
- Target recruitment for the round, overall and split by treatment group.
- Total recruitment to date for the round, overall and split by treatment group.
- Overall number of patients recruited by centre will be presented graphically and in a tabular format.
- Monthly recruitment numbers will be presented graphically, with cumulative and targeted monthly recruitment superimposed.

A CONSORT diagram, per round, will be used to summarise the number of patients who were randomised to each round, received allocated intervention, did not receive allocated intervention; lost to follow up, discontinued treatment; analysed and excluded from analysis. The number of patients screened and reasons why patients who are not subsequently randomised will also be presented where available. This will be produced for any main analyses.

3.2 RANDOMISATION

Patients in each round will be randomly assigned to receive either investigator choice therapy or novel therapy. Randomisation will be based upon a minimisation randomisation algorithm incorporating a random element; this method will stratify patients by the following:

- Planned control treatment regimen (RCVP vs RCHOP vs rituximab with bendamustine vs rituximab with lenalidomide vs obinutuzumab with bendamustine)
- High risk FL (Yes vs No), with high risk defined as either POD24 (progression of disease within 24 months of starting first line induction immunochemotherapy, without high-grade transformation) or failure to achieve CR/CMR at the end of first line induction immunochemotherapy.
- Consolidation intention (stem cell transplant vs maintenance antibody vs none)
- Prior lines of therapy (1 line vs >1 line)

The allocation ratio utilised within each round is as follows:
Round One – 1:1 Allocation
Round Two & Three – 4:1 Allocation in favour of the relevant experimental arm
A 20% random element will be implemented within all randomisation algorithms to ensure that the allocation of patients does not become predictable.

Information pertinent to randomisation will be reported in all closed reports produced, this will include summaries of the stratification variables split by treatment to ensure the randomisation algorithm is working as expected and a summary of the number of patients randomised to each treatment group.

3.3 INELIGIBLE PATIENTS

Ineligible patients are defined as those randomised patients who are subsequently found to not meet the eligibility criteria of the trial. The number of ineligible patients and reasons for their ineligibility will be reported; a sensitivity analysis may be conducted and reported if the number of ineligible patients is deemed to be substantial.

Ineligible patients that had sufficient treatment will be included within the mITT analysis population if the reason for ineligibility is not deemed to have an influence on the primary outcome measure. An example where the ineligibility may dictate that the patient would not be included in the population could be if a patient had transformed to high grade lymphoma.

4. DATA QUALITY

4.1 DATA VALIDATION

Statistical data validation checks will be carried out at least annually, or before any planned analysis. These checks will be carried out in accordance with the most up to date version of the data validation plan, held in the trial management folders. This validation will include, at a minimum, checks of all critical data items, checks that all dates are entered correctly and that all forms are present as expected. All identified queries will be reported to the trial coordinator to be addressed, with a record of the validation stored within the statistical trial master file (TMF).

4.2 CRF DATA CAPTURE

This trial will utilise electronic remote data capture (eRDC) allowing sites to directly enter patient data into the trial database. Certain CRF's including the eligibility and SAE form will require investigator review and sign off. The remotely entered data will be reviewed by the trial co-ordinator (or delegate) and queries raised where required. CRF return rates for each CRF will be calculated as the number and proportion of returned CRFs over the number of expected CRFs at the point of analysis.

Any forms with substantial missing data, or any forms that have poor return will be highlighted to the trial co-ordinator to be addressed.

4.3 LENGTH OF PATIENT FOLLOW UP

Following the end of treatment (for any reason) patients will be followed up annually until the end of the trial (a minimum of 3 years) for disease progression, initiation of further therapy, survival, and any significant treatment related adverse events. Quality of life questionnaires will be collected every 24 weeks in non-progressed patients. Patients lost to follow-up will not be excluded from the trial analysis but will be included with an appropriate censoring time.

Where relevant the following information will be reported for each round, split by treatment allocation:

- Median length of follow-up, calculated as time from randomisation to patient's date last seen, presented graphically using reverse Kaplan-Meier analysis.
- A date last seen graph will be presented, identifying the status of each patient in relation to the time since date of randomisation. Status will indicate whether patients are alive, dead or have withdrawn from the trial.
- The number of patients lost to follow-up will be reported, split by treatment arm, alongside the reason for loss if known.
- The number of patients withdrawn from the trial, including the level of withdrawal, reason for withdrawal and time from randomisation to withdrawal, will be presented using tabulations and line-listings.

5. TRIAL POPULATION

5.1 BASELINE PATIENT CHARACTERISTICS

5.1.1 ALL ROUNDS

For each round a descriptive comparison of patient's demographics and clinical baseline characteristics at trial entry will be presented, overall and split by treatment arm, in all reports produced. These characteristics will include, but will not be limited to, the stratification variables used within the minimisation algorithm, age, sex, disease stage, relevant haematological and biochemical assessments.

Where appropriate continuous variables will be presented graphically, otherwise means, SD and range if data are normal and median, IQR and range if data are skewed. For categorical variables, the number and percentage of patients in each category will be reported. No hypothesis testing will be conducted; any imbalance of clinical importance will be noted and reported upon.

5.1.2 ROUNDS TWO & THREE

As non-concurrent control patients from earlier rounds will be utilised in the analysis of the primary outcome for these two rounds, baseline characteristics of control patients from the relevant rounds need to be compared. This will not include hypothesis testing, but any differences in characteristics that may be deemed clinically to impact upon patients' response to treatment should be reported upon. If any such imbalances are found, consideration around the weighting used when borrowing patients' data for the primary analysis should be given. Any changes to the weighting should be made clear with sensitivity analyses conducted including what the outcome would have been if the pre-determined weight of 0.75 had been used. If the decision is taken not to reduce the weightings used despite any imbalances this needs to be clearly justified within the report with sensitivity analyses at lower weightings also reported upon.

If the baseline characteristics don't show any imbalance or change in patient demographics between the rounds, then this should be reported upon and the pre-determined weighting of 0.75 used within the primary outcome analysis.

The DMC should be involved in any decisions made regarding the weightings that will be used within any primary analyses for these rounds. The contents of this report are described in Section 2.3.2.

5.2 DEFINITION(S) OF POPULATIONS FOR ANALYSIS

5.2.1 MODIFIED INTENTION TO TREAT POPULATION

This population will include all patients recruited to the relevant round with patients analysed in the group to which they are allocated at randomisation, including any patients that discontinue treatment early or are found to be ineligible post randomisation where ineligibility is not deemed to impact patients' response to treatment (Section 3.3). Patients who undergo stem-cell transplant (SCT) within 24 weeks of randomisation, patients who fail to start treatment and patients whose ineligibility is deemed to impact upon response to treatment will be replaced. Any patients who are replaced will not be included within this population.

5.2.2 SAFETY POPULATION

This population will include all randomised patients who started trial treatment, including those replaced. The definition of starting treatment may differ between the round's dependent upon the novel or control treatment being given. The below table details for each treatment the definition for starting treatment.

Treatments	Definition of Starting
**Control**	
Rituximab and bendamustine	At least one dose of either rituximab or bendamustine.
Rituximab and CVP	At least one dose of rituximab, cyclophosphamide, vincristine, or prednisolone.
RCHOP	At least one dose of rituximab, cyclophosphamide, doxorubicin, vincristine, or prednisolone.
Rituximab and lenalidomide	At least one dose of either rituximab or lenalidomide.
Obinutuzumab and bendamustine	At least one dose of either Obinutuzumab or bendamustine.
**Experimental**	
Round One: Epcoritamab and lenalidomide	At least one dose of either epcoritamab or lenalidomide.
Round Two: TBC	
Round Three: TBC	

6. TREATMENT RECEIVED

To assess treatment compliance within each round of the trial the information detailed in this section will be reported and presented descriptively. Given the number of potential control treatments and the use of three different novel therapies this section has been split to show how each treatment will be reported upon.

6.1 ALL ROUNDS

For all three rounds of the trial the following overarching statistics will be reported:

- The number of patients who start treatment, overall and split by treatment arm.
- The number of patients randomised who did not start treatment, including the reason(s) for not starting treatment, split by treatment arm.
- The time from randomisation to start date of treatment, split by treatment arm.
- The number of early treatment discontinuations, overall and split by treatment arm. Including the reason(s) for discontinuation
- The treatment status of patients at the time of analysis, categorised as follows: on treatment, completed treatment and discontinued treatment. Presented overall and split by treatment group.

6.2 CONTROL TREATMENTS

This section will report upon the individual treatments received in the control arm; a tabulation will be provided indicating how many patients are reported to be receiving each of the 5 pre-defined treatments.

For each defined treatment the following will be reported:

- The maximum number of cycles received by each patient at the time of analysis.
- The number of dose modifications, alongside the reason(s) for dose modifications. Indicating which of the treatments within the combination, where applicable, was dose modified.
- The number of missed doses, which treatment within the combination was missed, alongside the reason(s) for the dose being missed.
- The number of treatment delays, the length of each delay and the reason(s) for any delays

6.3 NOVEL TREATMENTS

6.3.1 ROUND ONE: EPCORITAMAB + LENALIDOMIDE

For this round the following treatment information will be reported:

- The number of cycles received by each patient at the time of analysis.
- The number of lenalidomide dose modifications, alongside the reason(s) for dose modifications. *Note: Dose modifications for Epcoritamab are not permitted under protocol v1.0*
- The number of missed doses, which treatment within the combination was missed, alongside the reason(s) for the dose being missed.
- The number of treatment delays, the length of each delay and the reason(s) for any delays

6.3.2 ROUND TWO

To be updated once treatment comparison is known.

6.3.3 ROUND THREE

To be updated once treatment comparison is known.

7. TOXICITY AND SAFETY ANALYSIS

Toxicity data will be collected continuously throughout the trial and graded according to CTCAE version 5.0. Toxicity and safety data will be reported for all patients within the safety population as defined in Section 5.2.2, for patients who are replaced due to transplant, only safety events reported up until the point of transplant will be included in the main reports. Any events reported after these patients have been transplanted will be reported separately, within the appendices of all reports. Toxicity and safety data will also be reported for all patients randomised (this will be held in the appendix of all reports) and presented within all closed reports produced and at the final analysis. The following information will be presented for each round in the following way:

7.1 ALL ROUNDS

- The total number of adverse events, split by treatment and overall.
- The total number of patients who experience at least one adverse event, split by treatment and overall.
- The number and proportion of patients experiencing grade 3 or higher adverse events, split by treatment (where appropriate) and overall.
- Maximum grade of adverse event experienced by each patient, split by treatment (where appropriate) and overall.
- Relatedness of adverse events, split by treatment and overall.
- Line-listings detailing all reported adverse events, presenting the number of events (occurrences of each adverse event) and the number of patients affected split by CTCAE category, CTCAE event term, grade, and treatment (where appropriate)
- Line-listings detailing each reported adverse event containing the following, patient number; CTCAE category, CTCAE event term, grade, start date, ongoing, end date (where applicable), length of event (where applicable), relatedness, outcome, sequelae, and treatment. This will be presented in all reports, for DMC reports only events reported since the last snapshot will be contained in the main report, all other events will be reported in the appendix.
- The total number of serious adverse events, overall and split by treatment.
- The number and proportion of patients experiencing one or more serious adverse event, overall and split by treatment.
- Line-listings of all reported serious adverse events, details will include patient number, admitting event, other associated events; reason for reporting; outcome; sequelae; grade; grade of other associated events; categorisation, relatedness, length of event and treatment allocation.

7.2 NOVEL TREATMENTS

7.2.1 ROUND ONE: EPCORITAMAB + LENALIDOMIDE

- The total number of relevant adverse events of special interest reported.
- The total number (and proportion) of patients who experience at least one relevant adverse event of special interest.
- A tabulation showing the number (and proportion) of relevant adverse events of special interest split by severity.
- A tabulation presenting the number (and proportion) of relevant adverse events of special interest, split by type.

- Line-listings of all reported adverse events of special interest, including the following information, patient number; treatment allocation; event type; grade and length of event.

Note: as per the protocol all adverse events of special interests for this combination are linked to the use of epcoritamab. The defined events are as follows: Cytokine release syndrome (CRS) of any grade; immune effector cell-associated neurotoxicity syndrome (ICANS) of any grade; any suspected hemophagocytic lymphohistiocytosis (HLH); Clinical Tumour Lysis Syndrome (TLS) of any grade & Neutropenic sepsis of any grade.

8. ANALYSIS

All analysis will be carried out using the mITT population defined in Section 5.2.1 unless otherwise stated.

8.1 DEFINITION AND CALCULATION OF OUTCOME MEASURES

8.1.1 PRIMARY OUTCOME MEASURE

Complete metabolic response (CMR) assessed by PET-CT and defined by the Deauville 5-point scale and Lugano 2014 criteria at 24 weeks from the start of induction therapy. Patients who die from any cause or relapse/progress prior to this time-point will be considered non-responder. Patients who don't have a PET-CT scan within the protocol defined window (+/- 2 weeks) or withdraw from the trial prior to this time-point will be considered non outcome evaluable and reported as such. Patients who undergo stem-cell transplantation prior to the 24-week scan or fail to start treatment will be replaced and hence not included in the analysis of this outcome.

PET-CT scans taken at the end of induction (24 weeks) will be centrally reviewed at the Imaging Core Lab. This review will use the Lugano Criteria and results of this review will be recorded and inputted into the trial database. In instances where the central review and site review provide different tumour responses this will be queried, and the central review response will be used as patients' response for the purpose of analysis.

Percentage agreement between sites initial review and central review will be reported in all reports produced.

8.1.2 SECONDARY OUTCOME MEASURES

- Overall metabolic response (CMR + PMR) assessed by PET-CT and defined by the Deauville 5-point scale and Lugano 2014 criteria at 24 weeks from the start of induction therapy. Patients who die from any cause or relapse/progress prior to this time-point will be considered non-responders. Patients who don't have a PET-CT scan within the protocol defined window (+/- 2 weeks) or withdraw from the trial prior to this time-point will be considered non outcome evaluable and reported as such. Patients who undergo stem-cell transplantation prior to the 24-week scan or fail to start treatment will be replaced and hence not included in the analysis of this outcome.
- Progression-free survival, defined as the time from randomisation to the date of first relapse/progression or death from any cause. Patients who are alive and relapse/progression free at the time of analysis will be censored at their date last seen.
- Overall survival, defined as the time from randomisation to date of death from any cause. Patients who are alive at the time of analysis will be censored at their date last seen.

- Duration of response, defined as the time from first response assessed by PET-CT (CMR or PMR) to the date of first relapse/progression or death from any cause. Patients who are alive and relapse/progression free at the time of analysis will be censored at their date last seen.
- Duration of complete response, defined as the time from first complete response, assessed by PET-CT, to the date of first relapse/progression or death from any cause. Patients who are alive and relapse/progression free at the time of analysis will be censored at their date last seen.
- Time to next treatment, defined as the time from randomisation to the start date of next treatment for lymphoma. Patients who are responding (CMR or PMR) who receive consolidation radiotherapy will not be considered an event and will be censored at their date last seen if no other treatment for lymphoma is reported. Patients who die without having started next lymphoma treatment will be considered a competing risk at their date of death, and patients who are alive and have not started next lymphoma treatment at the time of analysis will be censored at their date last seen.
- Safety will be collected in accordance with CTCAE criteria v5.0 with toxicity defined to be any grade 3 or higher adverse event or a serious adverse event of any grade. Safety is monitored continuously throughout patients' treatment, with AE's collected until 60 days post final dose of IMP.
- Quality of life (measured using the EQ-5D-5L, EQ-VAS and FACT-Lym) collected pre-treatment, Cycle 3 Day 1, 24 weeks from the start of treatment and then every 24 weeks in the pre-progression follow-up until the end of study.

8.2 ANALYSIS OF PRIMARY OUTCOME MEASURES

CMR by PET-CT at 24 weeks will be reported as numbers and proportions, with the numerator the number of patients achieving a complete response and the denominator the total number of patients randomised to the relevant treatment group (including patients reported as being not evaluable but not including patients that are replaced). This will be tabulated by treatment group and response (CMR vs No CMR). In addition to this tabulation, Bayesian posterior probability plots will be presented alongside the probability that the true difference between the treatment arms surpasses a range of relevant thresholds (10%, 15% & 20%).

For the Round 1 analysis a minimally informative beta prior of Beta(1,1) will be employed. In subsequent rounds this prior will be informed by previous control group response rates and as such the prior and weightings utilised within these analyses will also be reported, future versions of this SAP will detail how the weightings employed will be determined.

Success, here being determination that a treatment should progress for further investigation, is defined to be finding a greater than 60% probability that the true difference between the experimental and the control arm is greater than 15%.

Prob(true difference between arms ≥15%)≥60%

8.2.1 SAMPLE SIZE DETERMINATIONS

Based on feasibility assessments a total sample size for all three rounds of 284 patients (95 control + 189 experimental arm patients in total) was deemed appropriate. Round (R)1 will treat 126 patients (63 patients per arm, 1:1 randomisation). R2 and R3 will treat 63 patients in the experimental arm and 16 in the control arm (4:1 randomisation). To make the most efficient use of available patients and reduce the number of control arm patients required in R2 and 3, data from patients recruited to previous control arms will be incorporated into subsequent rounds using power priors.

We performed sample size justifications using a Bayesian approach to calculate the probability that the PET-CT CMR rate in the experimental arm is greater than a given value. Due to the Bayesian approach taken no alpha or power is presented; instead, probability statements are used to evaluate the primary outcome.

Operating characteristics for the proposed design have been conducted to assess the probability of drawing the correct conclusions, under predefined conditions. The simulations conducted replicate the proposed design and cover four main scenarios; Scenario 1: We observe a higher response rate in the control group compared with the experimental arm; Scenario 2: We observed no difference between the repose rate in the control and experimental group; Scenario 3: We observe exactly a 15% difference in response rates (in favour of the experimental arm); and Scenario 4: We observe a larger than 15% difference in response rates (in favour of the experimental arm). Simulations for all three randomisations were conducted for control rates ranging between 40% and 60%, looking for a >15% difference under each of the four scenarios, with certainty levels ranging from 50% to 80% with operating characteristics produced from 10,000 simulations. For randomisations 2 and 3, simulations were conducted for each permutation of the preceding randomisation(s) observed control rate and the observed control rate in the current randomisation, applying weightings to previous data of 0.75 and 0.5. Results of some of these simulations are presented below, however a full simulation report can be found in the following location: T:\Trials Work\HAEMATOLOGY\New business\TAP\REFRACT\SAP\SimulationReport

For R1 we employed a conjugate Beta-Binomial analysis with a non-informative prior of Beta(1, 1). The probabilities presented below are based on the true difference in PET-CMR rate ≥15% (a difference of 15% is thought to be clinically meaningful) and calculations have been performed to detect this difference compared with control group PET-CMR rates of 40, 50 and 60%. Table 1 shows the probability that the true difference between treatment arms is ≥15% based on a 40% PET-CMR rate in the control group and 126 patients recruited (i.e., 26/63 responses). If we observe a difference of 20% between treatment arms there is a 67% probability that the true difference in response rates is ≥ 15%. If the observed difference is as high as 30% then the probability that the true difference is ≥15% will be 96%. In scenarios with a 50% PET-CMR rate in the control group, the probability that the true difference is ≥15% is 74% when a 20% difference is observed, as shown in Table 2.

Table 1. Probabilities of true difference being >15% based on 40% control rate.

Control (n=63)		Experimental (n=63)		Difference in PET-CMR rates (%)	Pr(true difference in RR≥0.15)
Responses	Response Rate (%)	Responses	Response Rate (%)		
26	40	26	40	0	0.04
26	40	32	50	10	0.26
26	40	38	60	20	0.67
26	40	45	70	30	0.96

Table 2. Probabilities of true difference being >15% based on 50% control rate.

Control (n=63)		Experimental (n=63)		Difference in PET-CMR rates (%)	Pr(true difference in RR≥0.15)
Responses	Response Rate (%)	Responses	Response Rate (%)		
32	50	32	50	0	0.04
32	50	38	60	10	0.26
32	50	45	70	20	0.74
32	50	51	80	30	0.97
32	50	57	90	40	1.00

All presented operating characteristics look at 60% certainty levels and apply a 0.75 weighting to incorporated data. For R1 we have presented operating characteristics for observed control rates of 40% and 60% (Table 3). For R2 we have presented the operating characteristics for observed R1 control rates of 40% and observed R2 control rates of 40% and 50%, and for observed R1 control rate of 60% and observed R2 control rates of 50% and 60% (Table 4). For R3 we have presented the operating characteristics for observed R1 control rates of 40%, observed R2 rates of 40% and 50% and observed R3 rates of 40% and 50%, as well as the operating characteristics for observed R1 control rate of 60%, observed R2 rates of 50% and 60% and observed R3 rates of 50% and 60% (Table 5).

Table 3. Round 1 operating characteristics

Control RR_{R1}	True Difference (Experimental RR_{R1} - Control RR_{R1})	Scenario			
		1	2	3	4
		-10%	0%	15%	25%
40%	Probability Positive Result	0	0.017	0.351	0.787
60%		0.001	0.018	0.367	0.844

Table 4. Round 2 operating characteristics

Control RR_{R1}	Control RR_{R2}	True Difference (Experimental RR_{R2} - Control RR_{R2})	Scenario			
			1	2	3	4
			-10%	0%	15%	25%
40%	40%	Probability Positive Result	0	0.017	0.385	0.818
	50%		0.008	0.12	0.722	0.971
60%	50%		0	0.001	0.113	0.509
	60%		0.001	0.021	0.384	0.859

Table 5. Round 3 operating characteristics

Control RR_{R1}	Control RR_{R2}	Control RR_{R3}	True Difference (Experimental RR_{R2} - Control RR_{R2})	Scenario			
				1	2	3	4
				-10%	0%	15%	25%
40%	40%	40%	Probability Positive Result	0	0.014	0.37	0.83
		50%		0.008	0.113	0.757	0.978
	50%	40%		0	0.01	0.308	0.771
		50%		0.003	0.08	0.687	0.966
60%	50%	50%		0	0.002	0.135	0.573
		60%		0	0.023	0.458	0.914
	60%	50%		0	0.001	0.092	0.496
		60%		0	0.015	0.376	0.874

8.3 ANALYSIS OF SECONDARY OUTCOME MEASURES

- Overall metabolic response rate at 24-weeks will be reported as number and proportions, with the numerator the number of patients who achieve a complete or partial metabolic response and the denominator the total number of patients randomised to the relevant treatment group (including patients reported as being not evaluable but not including patients who are replaced). This will be reported tabulated by treatment group and response (OMR

(CMR+PMR) vs No OMR) as well as a tabulation split by response at 24-weeks (CMR, PMR, SD, PR, NR, NE). A Chi Squared test or Fishers exact test will be used to compare between treatment arms as appropriate.
- OS, PFS, DoR and DoCR will be reported using Kaplan Meier curves with survival estimates at 12, 24 & 36 months presented alongside 95% confidence intervals (CI). A log rank test will be used to compare between treatment arms alongside a Cox regression model, which at a minimum will includes variables to represent the stratification factors.
- TTNT will be reported using cumulative incidence curves with estimates at 12, 24 & 36 months presented alongside 95% CIs. A Fine and Gray's test will be used to compare by treatment group.
- Safety will be reported as the number and proportion of patients who experience at least one adverse event, grade 3 or higher, or a serious adverse event of any grade. This will be reported tabulated by treatment arm and compared between treatment arms using Chi squared or Fishers exact tests as appropriate.
- Quality of life data will be scored as per the relevant scoring guides, EQ-5D-5L User guide for EQ-5D-5L and EQ-Vas scores and FACT-Lymphoma Scoring Guidelines (v.4) for the FACT-Lym. Patients' responses will be presented in tabular format reporting the number and proportion of patients at each level split by the dimensions, the symptom and function scores will be presented graphically. The overall scores from both questionnaires will be compared between arms and analysed using longitudinal methods.

8.4 ADDITIONAL ANALYSES

Sensitivity analyses will be carried out for Rounds 2 & 3. These analyses will assess the suitability of the applied weightings within the primary outcome. Re-analysing using lower weightings (e.g., 0.5 & 0.25 if 0.75 is the weighting applied in the main analysis) or higher weightings (e.g., 0.75 if 0.5 in the weighting applied in the main analysis)

An additional analysis of the primary outcome adjusted for the stratification factors will be conducted, this will utilise a Bayesian Logistic Regression model with power-priors built in for the relevant rounds.

8.5 SUBGROUP ANALYSIS

A sub-group analysis looking at patients with defined high-risk disease will be conducted, these analyses will compare the effect of treatment within these patients. Due to the potentially small groups stratifying by risk score may produce, and lack of power these analyses will be exploratory only and results treated with caution.

9. STATISTICAL SOFTWARE

All analysis will be carried out in appropriate statistical software: SAS 9.4, Stata 17, or R 4.0.2. Later versions of the programs may also be used.

10. STORAGE AND ARCHIVING

Raw data snapshots, analysis programs, outputs and other relevant documentation will be stored in the following location:
T:\Trials Work\HAEMATOLOGY\REFRACT

11. REFERENCES

Ibrahim JG, Chen MH, Gwon Y, Chen F. The power prior: theory and applications. Stat Med. 2015;34(28):3724-49.
